# Mechanistic Insights into the Electroreduction of Carbon Dioxide to Formate on Palladium

**DOI:** 10.1021/acscatal.5c04052

**Published:** 2025-09-25

**Authors:** Maximilian Winzely, Deema Balalta, Adam H. Clark, Tommaso Iarocci, Paul M. Leidinger, Davide Masiello, Meriem Fikry, Tym de Wild, Maximilian Georgi, Sara Bals, Thomas J. Schmidt, Juan Herranz

**Affiliations:** † PSI Center for Energy and Environmental Science, CH-5232 Villigen PSI, Switzerland; ‡ Electron Microscopy for Materials Science, University of Antwerp, BE-2020 Antwerpen, Belgium; § PSI Center for Photon Science, CH-5232 Villigen PSI, Switzerland; ∥ Physical Chemistry, Technische Universität Dresden, DE-01062 Dresden, Germany; ⊥ ETH Zürich, Institute for Molecular Physical Science, CH-8093 Zürich, Switzerland

**Keywords:** CO_2_RR, Palladium, Palladium Hydride, Formate, CO poisoning, GIXAS, ATR-SEIRAS

## Abstract

The electrochemical reduction of carbon dioxide (or the CO_2_-reduction reaction, CO_2_RR) presents a promising strategy to mitigate CO_2_ emissions while producing valuable chemical feedstocks. Palladium (Pd) catalysts are particularly interesting for their capacity to selectively produce formate at low overpotentials and carbon monoxide (CO) at higher overpotentials. However, palladium’s CO_2_-to-formate activity is often hindered by the progressive poisoning of its surface with CO. To shed light on the parameters that control this performance-determining process, in this study we employ Operando grazing incidence X-ray absorption spectroscopy and attenuated total reflectance surface-enhanced infrared absorption spectroscopy to investigate the CO_2_RR mechanism on carbon-supported Pd nanoparticles (Pd/C) and a freestanding Pd aerogel with similar electrochemical surface areas but substantial differences in hydride formation, CO poisoning, and catalytic performance. Pd/C demonstrates rapid hydride formation and constant formate activity at −100 and −200 mV vs the reversible hydrogen electrode, revealing an indirect correlation between activity for formate and hydride stoichiometry that strongly indicates the active involvement of the surface hydride in the CO_2_RR to formate. In contrast, the Pd aerogel suffers from rapid CO surface poisoning and a concomitantly negligible formate-production activity at the same potentials. These differences in catalytic behavior are linked to an increased presence of grain boundaries in the aerogel’s surface that has been tied to a reduction in the activation barrier for CO_2_ conversion to the surface-adsorbed *COOH and the subsequent formation of strongly adsorbed CO. As such, our findings highlight how optimizing the structural features of Pd-based surfaces can lead to significant enhancements in their efficiency toward formate production.

## Graphical abstract

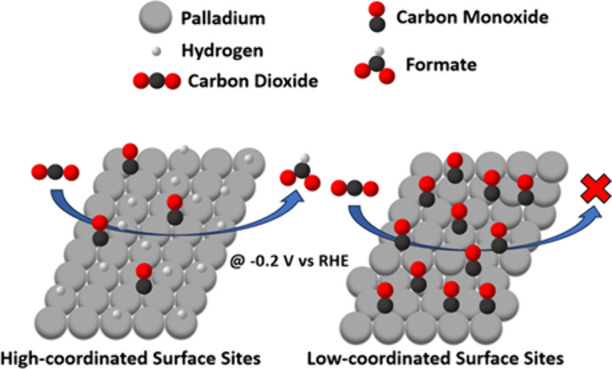

## Introduction

With global warming accelerating at an unprecedented rate,^1^ it is crucial to develop cost-effective techniques to reduce the volume of the ever-increasing emissions of carbon dioxide (CO_2_) going into the atmosphere. One promising approach is the introduction of CO_2_ utilization technologies that convert CO_2_ into valuable chemicals that can then serve as industrial feedstocks. Among these methods, the electrochemical reduction of CO_2_ (or CO_2_-reduction reaction, CO_2_RR) has attracted a lot of attention in recent years, especially when the target products are formate or carbon monoxide (CO), since these two compounds have been regarded as economically profitable when produced through this approach.^2–5^

In this context, palladium (Pd) stands out as an exceptional catalyst thanks to its unique ability to selectively produce formate or CO at low vs high potentials (i.e., at ≈0.1 to ≈ −0.4 vs ≈ −0.5 to ≈ −0.9 V vs the reversible hydrogen electrode (RHE), respectively).^6–13^ In addition, Pd is also singular among the transition metals because it forms a hydride phase (PdH_*x*_) at the potentials at which the CO_2_RR takes place.^14,15^ In general, PdH_*x*_ can exist either as an α-phase with a hydrogen-to-Pd ratio (*x*) of ≈ 0.05, or as a β-phase characterized by *x*-values ≥ 0.5 (whereby the exact values are strongly influenced by the Pd-particle size). A mixed phase consisting of both α- and β-PdH_*x*_ exists between these two stoichiometric limits.^16–20^ Moreover, while in CO_2_-free electrolytes the α- phase starts to form at ≈300 mV vs RHE and the β-hydride at ≈50 mV vs RHE,^21–25^ their formation becomes more complex in CO_2_-saturated electrolytes, as evidenced by the considerable discrepancies in the literature regarding the potential of β-hydride formation^26^ (with reported values ranging from −0.1 to −0.8 V vs RHE).^11–13,27–30^ In a recent study, we have shown that these discrepancies possibly stem from the additional formation of a surface-adsorbed CO layer at all CO_2_RR-relevant potentials, which competes with the adsorption of hydrogen atoms on the catalyst surface and the subsequent formation of PdH_*x*_, and is both potential- and time-dependent.^26^

Beyond these discrepancies, several studies agree that the hydride phase plays a crucial role in the CO_2_RR-mechanism toward formate on Pd, even if it remains controversial which hydride phase is active and how exactly it partakes in the mechanism. Gao et al.^6^ and Abdellah et al.^30^ suggested that a mixed α/β-hydride phase is responsible for CO_2_ reduction to formate, while Min et al.^7^ and Rahaman et al.^10^ proposed that it is the β-phase (which in their case forms at high potentials) that is active. Furthermore, Min et al.^7^ showed that the poisoning of the Pd-surface with CO progressively decreases formate production, a phenomenon that we also observed in a recent *Operando* IR-spectroscopy study, and that emphasizes the complexity of this catalytic system.

To better understand how the hydride phase participates in the mechanism of the CO_2_RR toward formate, in this work we have used *operando* grazing incidence X-ray absorption spectroscopy (GIXAS)^21^ and attenuated total reflectance surface-enhanced infrared absorption spectroscopy (ATR-SEIRAS) to study carbon-supported Pd nanoparticles (Pd/C) and a freestanding Pd aerogel with similar electrochemical surface areas (ECSAs) but high vs low CO_2_-to-formate currents, respectively. Chiefly, we have done so using novel flow cells that enable the use of low Pd-loadings entailing catalyst layers (CLs) < 2 μm in thickness (and thus devoid of mass transport limitations along the CL-thickness)^31–33^ and connected to an analytical setup that permits the direct correlation of time-resolved PdH_*x*_ formation and CO-adsorption with the changes in CO_2_RR-product distribution. Our results demonstrate that lattice strain within the Pd catalyst causes severe CO poisoning, which significantly suppresses formate production in the low overpotential range (−0.1 to −0.4 V vs RHE).

## Experimental Section

### Pd Aerogel Synthesis

The Pd aerogel was synthesized following the methods of Georgi et al.^34^ and Liu et al.^35^ First, 1.6 mL of a 50 mM ethanolic stock solution of PdCl_2_ (Alfa Aesar, 99.999%) was diluted in 390 mL of denatured ethanol (Berkel AHK, 99%, 1% petroleum ether) at room temperature. Subsequently, 9.58 mL of a freshly prepared 50 mM sodium borohydride solution (NaBH_4_, Sigma-Aldrich, 99.99%, granular) was rapidly added with vigorous stirring (450 rpm) until a molar ratio of reducing agent to Pd^2+^ ions (n_H^–^_: n_Pd^2+^_) of 12 was obtained. The mixture was stirred for a further 30 s and then left at room temperature for 16 h. The resulting black gel precipitate was collected and washed at least eight times with ethanol. Finally, the resulting solvogels were supercritically dried with CO_2_ at 45 °C and 105 bar in an autoclave (13200J0AB, Spi Supplies).

### Electrode and Electrolyte Preparation

The working electrodes for the spectroelectrochemical GIXAS flow cell were prepared by drop-casting the catalyst onto a 35 μm thick graphene sheet (Nanografi) that was used as the working electrode substrate. For this, an ink was prepared by first weighing ≈5 mg of the 40 wt % Pd/C (Premetek, P30A400) or ≈1 mg in the case of the Pd aerogel into a glass vial. For the Pd/C catalyst ultrapure water (18.2 MΩ·cm – Elga PureLab) and isopropanol (Sigma-Aldrich, HPLC grade, 99.9%) were added in a volume ratio of 3 to 7, whereby the total solvent volume was calculated to yield a loading of 100 μg_Pd_/cm^2^ when using a droplet volume of 20 μL. In contrast, for the Pd aerogel only ultrapure water was added, and the volume was adjusted to yield a loading of 50 μg_Pd_/cm^2^ with a droplet volume of 35 μL. Nafion resin was added as a binder with a carbon-to-binder ratio of 1:0.2 for Pd/C and a catalyst-to-binder ratio of 1:0.1 for the Pd aerogel. Both inks were subsequently sonicated for 10 vs 1 min for Pd/C vs the Pd aerogel, respectively. For a precise and reproducible positioning of the catalyst layer onto the graphene sheet, the same mask used in our previous studies was employed.^21^

The counter electrode was prepared by spray-coating carbon black as a sacrificial material on a conductive Kapton film substrate with an additional gold layer of 50–100 nm sputtered on top. For this, an ink of Black Pearls 2000 was prepared by a weighed amount of this carbon black with ultrapure water and isopropanol in a volume ratio of 7:3 along with the amount of Nafion binder needed to attain a carbon-to-ionomer ratio of 1:0.44. The volume of the solvent mixture was adjusted so that a carbon concentration of 5 mg/mL was achieved. After sonication for at least 20 min, the ink was automatically sprayed onto pieces of the gold-sputtered Kapton foil that were secured in a custom-made mask that delineated an active area of ≈0.5 cm^2^ for a circular electrode with a diameter of 8 mm. The final carbon loading on each substrate was ≈0.6 mg_C_/cm^2^, as measured with a microbalance (Mettler Toledo XPE206DR) and using a dummy substrate method described in our previous work.^36,37^

For the *operando* ATR SEIRAS studies both catalysts were again drop-casted onto an Au-coated silicone ATR crystal by using an ink. The ink formulation stayed the same as above, with the difference that for Pd/C the total volume of the ink was adjusted so that a droplet of 60 μL yielded a loading of 25 μg_Pd_/cm^2^. In this case the drop-casting procedure was repeated 3 times to achieve a final loading of 75 μg_Pd_/cm^2^ for the Pd/C working electrode. For the Pd aerogel, the ink volume was adjusted so that a droplet of 120 μL resulted in a catalyst loading of 25 μg_Pd_/cm^2^. To position the catalyst layer within the correct position on the ATR crystal a drop-casting setup which was described in one of our previous studies was employed.^38^ In the case of the Pd/C catalyst the drop-casting setup was heated up to ≈70 °C and the ink was dried with a hot air stream. For the Pd aerogel the drop-casting setup was put under vacuum (≈30 mbar) by placing it into desiccator.

For the used phosphate buffer, 1.872 g of dipotassium hydrogen phosphate (K_2_HPO_4_, Merck LiChropur, anhydrous, 99.999%) and 1.939 g of potassium dihydrogen phosphate (KH_2_PO_4_, Merck, LiChropur, anhydrous, 99.999%) were dissolved in 250 mL of ultrapure water (18.2 MΩ·cm, ELGA Purelab Ultra) to achieve a 0.1 M concentration. In the case of the 0.1 M KHCO_3_ solution, 2.513 g potassium bicarbonate (99.95% trace metal base, Sigma-Aldrich) was dissolved in 250 mL ultrapure water.

### Electrochemical Procedures

The Ag/AgCl reference electrode was calibrated against the RHE scale by performing separate hydrogen evolution/oxidation experiments with a platinum rotating disk electrode in a H_2_-saturated 0.1 M phosphate buffer with the same pH as the CO_2_-saturated 0.1 M KHCO_3_ electrolyte.

For the CO_2_RR experiments, the 0.1 M KHCO_3_ electrolyte was presaturated for at least 15 min before each test by bubbling CO_2_ directly into the corresponding electrolyte bottle. Once the electrolyte was saturated the cell compartments were filled at a flow rate of 2 mL/min. The first electrochemical procedure was to record an impedance spectrum to determine the high frequency resistance which resulted repeatedly in a value of ≈35 Ω·cm^2^ for the GIXAS flow cell and of ≈100 Ω·cm^2^ for the ATR SEIRAS flow cell. In the following all the applied potentials were corrected for 85% of these resistances. Next, the working electrode was conditioned by recording 10 cyclic voltammograms (CVs) between −0.05 and 1.30 V vs RHE at 100 mV/s, followed by 10 CVs at 100 mV/s, 5 CVs at 50 mV/s and 2 CVs at 20 mV/s between 0.05 and 1.2 V vs RHE.

To the outlet of the working electrode chamber of the spectroelectrochemical GIXAS and ATR-SEIRAs flow cells were connected to an analytical setup that allows the time-resolved detection and quantification of gaseous products (e.g., hydrogen and carbon monoxide) by means of gas chromatography and mass spectrometry, and also of liquid products (e.g., formate) by ion chromatography. More details about these setups can be found in our two previous publications.^21,38^

### *Operando* XAS

For the *Operando* XAS experiments, we used an updated version of our spectroelectrochemical GIXAS flow cell including improvements featured by the ATR-SEIRAS cell^38^ introduced in our previous work. The primary update is presented in Figure S1 and consists of an improved flow field designed to enhance the mass transport properties of reactants to the catalyst layer and the removal of products. In particular, the thickness of the electrolyte layer was set to ≈200 μm by machining an extrusion on the inner counter-electrode parts (element 6 in Figure S1) over which the membrane (8) separating the working and counter electrode compartment is stretched. When the cell is sealed this extrusion extends into the working electrode gasket (11). In addition, a 200 μm thick polytetrafluoroethylene flow field (12) was added between the working electrode assembly (13) and the membrane (8) to ensure a controlled electrolyte layer thickness of 200 μm. In contrast to the original design of this spectroelectrochemical cell,^21^ the geometrical area of the catalyst layer has been reduced and now forms a circle with a diameter of 6 mm instead of the previous 10 mm. It is expected that this change further improves the mass transport properties, as the electrolyte flow should be faster in the center of the flow field, while the speed decreases toward the edges.^39^ For a better understanding of the flow field design, we have included a video of the cell assembly in the Supporting Information. The position of the reference electrode (7) has also been adjusted, so that it is now inserted into the counter electrode compartment (6) at an angle that positions the tip of the electrode closer to the membrane (8) and thereby further away from the counter electrode (5), where strong gas evolution could otherwise lead to a loss of potential control due to bubble accumulation on the electrode. A further optimization to improve the X-ray transmittance was made by adding a second slit in the outer working electrode part (15). The slit was mirrored to reduce the amount of polyether ether ketone (PEEK) obstructing the beam path and thus to minimize potential absorption of the X-ray beam. In addition, as described for our ATR-SEIRAS cell,^38^ a peristaltic pump with integrated pulsation dampeners was used to ensure a more stable and uniform electrolyte flow rate during the electrochemical experiments (when compared to the syringe pump used for this purpose in our previous study^21^). To illustrate the mass transport properties of the updated flow field, ORR experiments were conducted on a Pt-sputtered working electrode to quantify the diffusion boundary layer thickness at various flow rates. The results, along with a detailed experimental explanation, are provided in Figure S2 of the Supporting Information. including a detailed description of the applied procedures and a comparison to the results of similar measurements performed with the ATR-SEIRAS cell.

The *Operando* XAS measurements were carried out at the SuperXAS beamline of the Swiss Light Source. During the electrochemical experiments, XA-spectra were recorded at the Pd-K edge (24,350 keV) in fluorescence mode. A polychromatic beam was generated with 2.9 T superbend magnets and collimated by a Pt-coated mirror at an angle of 2.84 mrad. The monochromatic beam was then generated using a liquid nitrogen cooled Si(111) channel-cut quick-scanning monochromator. For optimal alignment in the grazing incidence (GI) configuration, a Pt-coated ring mirror focused the beam onto a 0.15 × 0.15 mm^2^ spot, resulting in a flux of 5 × 10^10^ photons/s. Three 15 cm ionization chambers filled with 1 bar Ar and 1 bar N_2_ were used to monitor the beam intensity. With the first two chambers, the intensity was measured before and after the sample interaction, while a Pd foil in front of the third chamber was mounted for energy calibration. Fluorescence detection was performed in quick XAS mode using a PIPS diode detector from Mirion Technology and 1 Hz oscillation of the monochromator. To precisely align the cell in the GI configuration, vertical, horizontal and angular scans were performed iteratively using the stage available at the SuperXAS beamline. This alignment was refined until the highest Pd K_α_ fluorescence signal was detected with the silicone drift detector.

For data treatment and analysis ProQEXAFS^40^ and Demeter software^41^ were used. For the EXAFS fitting the crystal parameters of Pd were extracted from a crystal structure file from the ICSD database for inorganic crystal structures (ICSD-257579). All the spectra were fitted in a *k*-range of 3–11.5 Å^–1^ and an *R*-range of 1–3.2 Å. For the fitting of the reference foil the coordination number was fixed to 12 to extract an amplitude reduction factor of 0.770 ± 0.038 (see Figure S3 and Table S1). The PdH_*x*_ stochiometry was determined by the following equation:^22,42^

$$\frac{\Delta R\left(T\right)}{R_{0}\left(T\right)}=0.0666x-0.0164x^{2}$$ 1

where *R*_0_(*T*) is defined as the Pd–Pd bond distance in the metallic state and Δ*R*(*T*) as the deviation from the latter caused by the (partial) formation of the corresponding PdH_*x*_ phase (in both cases at the given temperature, *T*).

For the time-resolved data during the potential hold studies, 120 spectra were averaged to produce one spectrum per 1 min. In the case of the linear sweep voltammetry (LSV) data an average of 50 spectra was used, which results in a potential resolution of 25 mV per spectrum considering the scan rate of 1 mV/s used to record the LSV. These spectra were linear combination fitted (LCF) in the next step, using the spectra of the Pd/C or the Pd aerogel at 500 mV and at −800 mV vs RHE as standards for the metallic and highest hydride states, respectively. Finally, the concentration profile obtained from the LCF was multiplied with the hydride stoichiometry for both catalysts at −800 mV vs RHE to yield the hydride stoichiometry of both catalysts at every presented data point.

### *Operando* ATR SEIRAS

In all ATR-SEIRAS spectroelectrochemical experiments, a 20 × 20 × 2 mm Au-coated silicon crystal with an ATR angle of 45 deg was used. For Au-coating, the chemical deposition procedure reported in our recent study was applied.^38^

ATR-SEIRAS measurements were performed using a Bruker FTIR spectrometer equipped with a liquid nitrogen-cooled mercury cadmium telluride detector. The spectral resolution was set to 4 cm^–1^, with a scan rate of 40 kHz. Each spectrum was produced by averaging 256 interferograms, resulting in a total acquisition time of approximately 1 min per spectrum. During the measurements, the sample chamber was held under vacuum (≈2 mbar). Before each potential hold a background spectrum was recorded at 500 mV vs RHE.

### Powder X-ray Diffraction (pXRD)

The pXRD measurements were performed with a SmartLab Rigaku system covering 2θ angles from 5 to 90°. A copper rotating anode (Cu K_α_ = 1.5406 Å) coupled with a Cu K_β_ filter was used in combination with a Rigaku HyPix3000 detector. All measurements were performed in the Bragg–Brentano configuration with the Cu source set to 200 mA and 45 kV for the Pd Aerogel and to 160 mA and 40 kV for the Pd/C catalyst.

### High-Angle Annular Dark-Field Scanning Transmission Electron Microscopy (HAADF-STEM)

HAADF-STEM was performed *ex situ* before electrochemical measurements using an aberration-corrected ThermoFisher Scientific Titan transmission electron microscope operating at 300 kV. The Pd/C powder samples were dispersed in ethanol, while the Pd aerogel was dispersed in Milli-Q water. Both suspensions were sonicated for less than 1 min and then drop-cast onto lacy carbon TEM grids for imaging.

## Results and Discussion

### Tracking PdH_*x*_ Formation through *Operando* GIXAS

As already discussed in the introduction, the main goal of this work is to gain a better understanding of the role of PdH_*x*_ in the mechanism of the CO_2_RR toward formate. For this purpose, the behavior of both Pd catalysts in the course of constant potential holds within the formate-producing potential regime was first investigated employing *operando* GIXAS with our novel spectroelectrochemical cell (see Figure S1). The formation of PdH_*x*_ over constant potential holds via LCF of the recorded XA-spectra, with a time resolution of 1 min per data point. For this LCF analysis, both electrodes were polarized at 0.5 V vs RHE for 10 min, and the averaged spectra were used as the standards for metallic Pd; complementarily, the standard spectrum for PdH_*x*_ with the highest hydride stoichiometry was derived by polarizing the electrodes of both catalysts at −0.8 V vs RHE for 45 min, with the final 5 min of data averaged to serve as the hydride standard. The bonding distances extracted from EXAFS fitting of the metallic and hydride standards (see Figures S4 and S5, along with Tables S2 and S3) were used in eq 1 to calculate the corresponding hydride stoichiometries, yielding *x* = 0.75 for Pd/C and *x* = 0.73 for the Pd aerogel. This hydride stoichiometry was then multiplied by the LCF component fraction of the hydride standard at each data point to convert the LCF results into time-dependent hydride stoichiometries. Finally, formate production was quantified every 6.5 min, while hydrogen evolution was continuously tracked via mass spectrometry with a dwell time of one second.

Focusing first on the results acquired on the Pd/C catalyst, Figure 1a shows the rapid formation (i.e., within ≤ 10 min) of PdH_*x*_ to equilibrium values across all investigated potentials, whereby the stoichiometries range from *x* ≈ 0.55 at −200 mV vs RHE to *x* ≈ 0.65 at −400 mV vs RHE. These stoichiometries are consistent with the results of Diercks et al.,^31^ who reported an x value of ≈0.6 for the full β-PdH_*x*_ formation for the same catalyst in CO_2_-free phosphate buffer at a higher potential of −100 mV vs RHE via *in situ* XAS measurements. Thus, full β-PdH_*x*_ formation appears to occur at all potentials with the exception of the hold at −200 mV vs RHE, for which the slightly lower value of *x* ≈ 0.55 suggests a possible mixed α + β phase.

**Figure 1:**
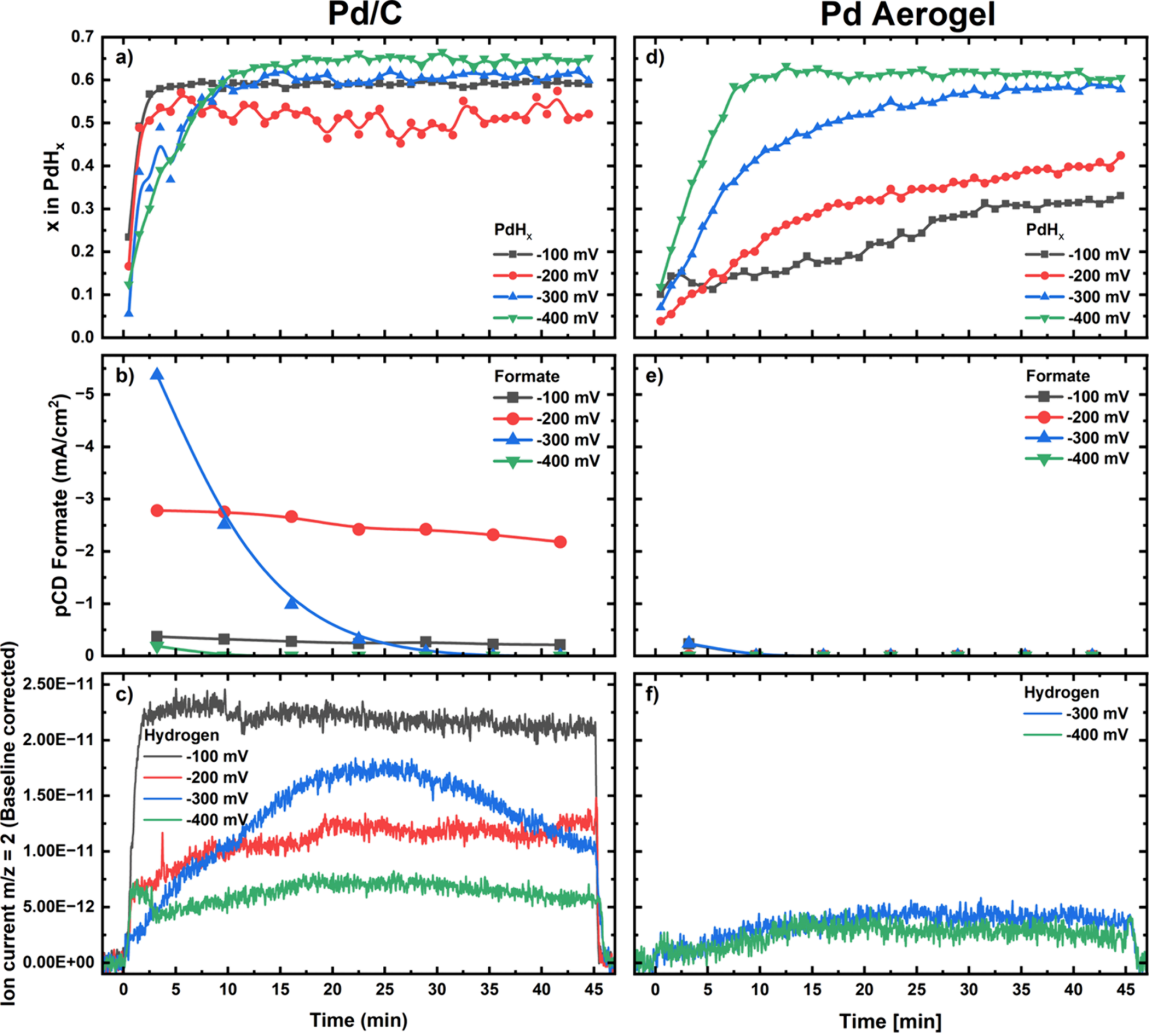
Time- and potential-dependent PdH_*x*_ stoichiometries (a, d), partial current densities (pCDs) for formate production (b, e) and ion current signals from the *online* mass spectrometer for an *m*/*z* = 2 (i.e., assignable to H_2_ – c, f) recorded on a Pd/C electrode with a Pd loading of 100 μg_Pd_/cm^2^ (a, b, c) or on a Pd aerogel electrode with a Pd loading of 50 μg_Pd_/cm^2^ (d, e, f), in CO_2_-saturated 0.1 M KHCO_3_ during 45 min potentials hold at −100, −200, −300, and −400 mV vs RHE.

When comparing the hydride formation results with the CO_2_-to-formate activity (expressed as a partial current density, pCD) in Figure 1b, a higher formate production is observed at −200 vs −100 mV vs RHE. The fact that the former potential entails a lower extent of hydride formation and a greater formate yield suggests that PdH_*x*_ might directly participate in the reaction mechanism, since the lower equilibrium PdH_*x*_ stoichiometry could stem from a higher consumption of surface hydrogen when more CO_2_ gets reduced into formate. Notably, the diminished H-content of the hydride phase at −200 mV vs RHE is confirmed by the EXAFS fitting of the XA-spectra averaged over the last 5 min of this and the other potential holds (see Figure S4 and Table S2), which yield the Pd–Pd bonding distances featured in Figure S6 in which the EXAFS at −200 mV vs RHE features an unambiguously shorter bond.

Complementarily to the above observations, the hydrogen evolution currents also correlate inversely with formate production, with more H_2_ being produced at −100 vs −200 mV vs RHE (see Figure 1b and c).^38^ Additionally, a significant decline in formate production and a steady increase in hydrogen partial current over time are observed at −300 mV vs RHE, again pointing at a strong link between surface hydride formation, formate and molecular hydrogen production. Specifically, when formate production decreases, more surface-adsorbed hydrogen appears to be available to combine into molecular hydrogen and/or to diffuse into the nanoparticles and form PdH_*x*_, highlighting the complex interplay between these processes and corresponding reaction products.

Interestingly, the results for the Pd aerogel reveal a significantly different behavior compared to the Pd/C catalyst (see Figure 1d–f). Notably, only very low formate production currents were observed at the beginning of the potential holds at −100 and −300 mV vs RHE (Figure 1d), while hydrogen evolution occurred exclusively at −300 and −400 mV vs RHE (Figure 1f). Moreover, while for the Pd/C catalyst PdH_*x*_ formed rapidly at all investigated potentials (i.e., equilibrated hydride stoichiometries were reached within the first ≈ 10 min of the holds), the Pd aerogel exhibited much slower hydride formation kinetics, as shown in Figure 1d. Particularly at −100 and −200 mV vs RHE, no equilibrated hydride stoichiometries were reached over the 45 min potential holds, suggesting a notable deficiency in hydrogen uptake for the aerogel compared to Pd/C. At −400 mV vs RHE, however, both catalysts exhibited a similar behavior, with hydride formation reaching an equilibrium stoichiometry of *x* ≈ 0.65 after ≈ 10 min and formate production remaining below the detection limits (except for the low activity observed at the beginning of the hold on the Pd/C catalyst).

To better understand the origins of these differences in behavior between Pd/C and the Pd aerogel, LSVs were recorded at the end of each potential hold starting from the corresponding potential (i.e., −100 to −400 mV vs RHE) and up to 1.25 V vs RHE, using scan rates of 20 mV/s up to −100 mV vs RHE and of 1 mV/s from that point on. Most importantly, this was done while keeping on recording XA-spectra. The corresponding results in Figure 2 display the hydride stoichiometry and current density during the LSVs for both catalysts. Focusing first on the Pd/C catalyst, a similar behavior was observed for the scans initiated at −100 and −200 mV vs RHE, with the XAS data in Figure 2a revealing a sharp onset of hydride deloading at ≈ 0.15 V vs RHE that triggers the oxidative current peaking at the same potential in Figure 2b. Additionally, the distinct oxidative current peaks observed at ≈ 0.85 V vs RHE can be assigned to the electrochemical stripping of CO adsorbed on the Pd-surfaces during the CO_2_RR potential holds,^26^ whereby the qualitatively greater charge and shift to higher potentials for the peak corresponding to the hold at −200 mV vs RHE suggest a larger CO coverage (further discussed below) and stronger binding to the Pd-surface at this lower potential.

**Figure 2:**
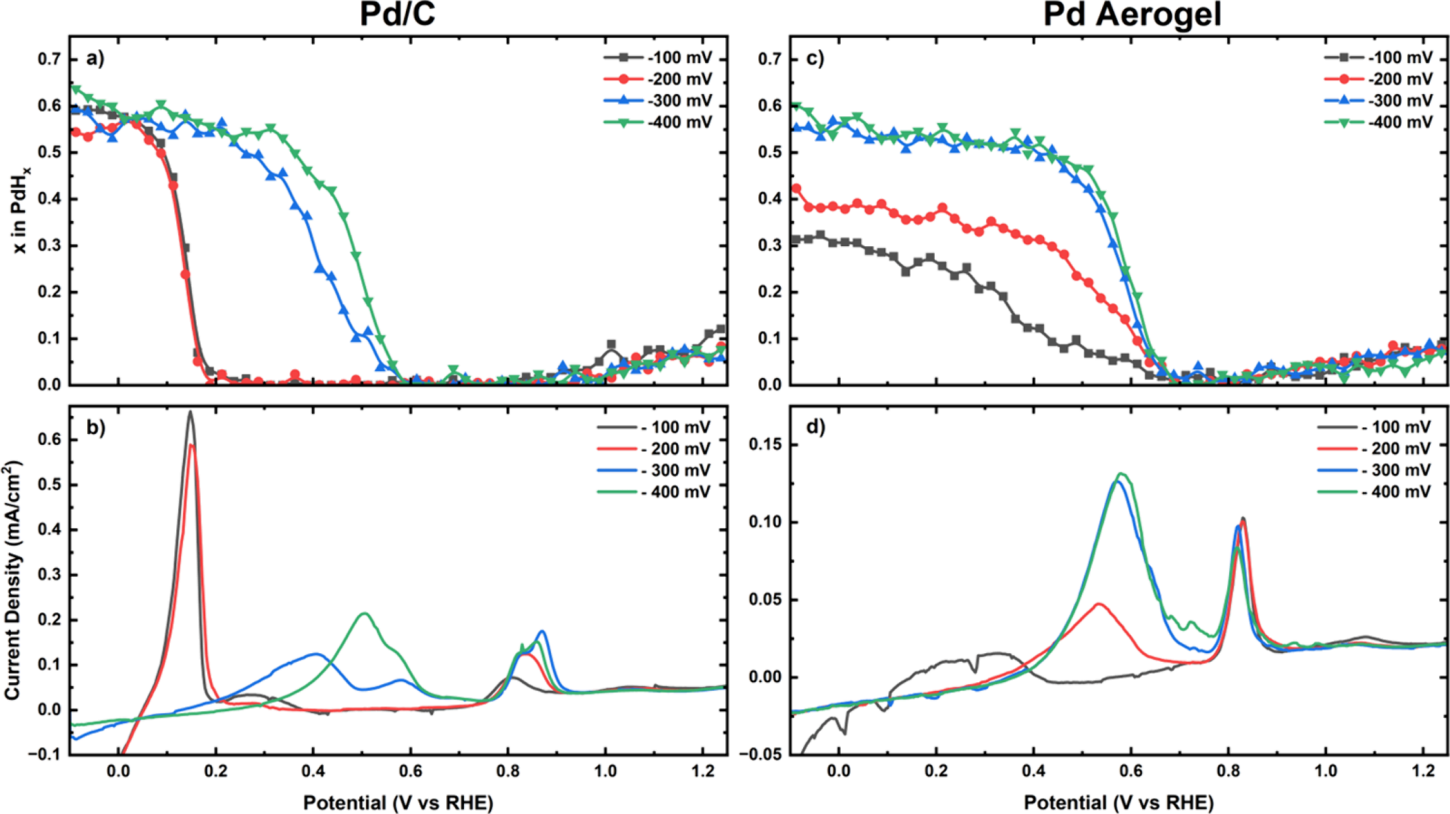
XAS-derived PdH_*x*_ stoichiometries (a, c) and corresponding current densities (b, d) recorded in CO_2_-saturated 0.1 M KHCO_3_ on a Pd/C electrode with a loading of 100 μg_Pd_/cm^2^ (a, b) or on a Pd aerogel electrode with a loading of 50 μg_Pd_/cm^2^ (c, d) during LSVs following 45 min potential holds at −100, −200, −300 or −400 mV vs RHE. The LSVs were recorded at a scan rate of 20 mV/s from the holding potential up to −100 mV vs RHE (not shown), and at 1 mV/s from −100 mV to 1.25 V vs RHE.

Turning our attention to the LSV recorded after the potential holds at potentials of −300 and −400 mV vs RHE entailing a significant decline in formate activity (see Figure 1a), the corresponding hydride oxidation currents shifted to higher potentials of ≈0.4 and ≈0.5 V vs RHE, respectively, and featured two peaks that are likely attributable to a polydisperse size distribution of the Pd-nanoparticles.^43^ Complementarily, the CO stripping currents display larger charges (vide infra)

As for the Pd aerogel, hydride deloading/oxidation systematically took place at higher potentials across all four LSVs, with complete hydride oxidation peaking at ≈0.55–0.6 V vs RHE with the exception of the measurement following the hold at −100 mV vs RHE (see Figure 2c and d). Moreover, unlike the Pd/C catalyst, no significant increase of the CO-stripping charges and minimal changes in the peak positions are observed between the various LSVs recorded on the Pd aerogel (Figure 2d), possibly indicating that a full CO adsorption layer has already formed at all investigated potentials for this material.

To shed light on these apparent differences in the extent of CO poisoning for each catalyst in a quantitative manner, an additional set of LSV experiments was conducted in the laboratory (i.e., without recording XA-spectra) in which the potential was scanned from the same hold values up to 1.25 V vs RHE at a scan rate of 20 mV/s following hold times of 1, 5, 10, and 45 min. The corresponding LSVs featured in Figures S7 and S8 (for Pd/C vs Pd aerogel, respectively) were baseline-subtracted using the CVs recorded after each sweep, and the areas (A_CO‑Stripping_) under the higher potential oxidative peak were integrated and related to CO-coverages using the following equation:

θ=ACO−StrippingνQCO−Stripping−θ=1 2

where *ν* is the scan rate used for recording the LSV and *Q*_CO‑Stripping‑θ=1_ represents the charge required to oxidize a full monolayer of CO adsorbed on the catalyst surface. This charge was determined from CVs recorded on each catalysts after holding the potential at −400 mV vs RHE for 45 min.

The resulting CO-coverages for both catalysts as a function of time are presented in Figure 3, which unveils clear differences in CO poisoning behavior between the two catalysts, particularly in the higher potential regime. For the Pd/C catalyst, full surface coverage with CO was not reached at potentials of −100 and −200 mV vs RHE (Figure 3a) within 45 min at which a constant rate of formate production was maintained (see Figure 1a). In contrast, the Pd aerogel achieved complete CO coverage by the end of the hold at −100 mV vs RHE and was fully poisoned almost from the first recorded date point (1 min) at −200 mV vs RHE (Figure 3b), agreeing with the negligible formate yield observed at this potential (see Figure 1e). At a lower potential of −300 mV vs RHE, the Pd/C catalyst reached full CO coverage after 10 min, providing an explanation for the observed decline in formate activity during the potential hold (Figure 1b). Finally, at the lowest potential of −400 mV vs RHE, both catalysts exhibited complete CO coverage from the start of the hold, explaining their similar CO_2_-to-formate performances (Figure 1b and e).

**Figure 3:**
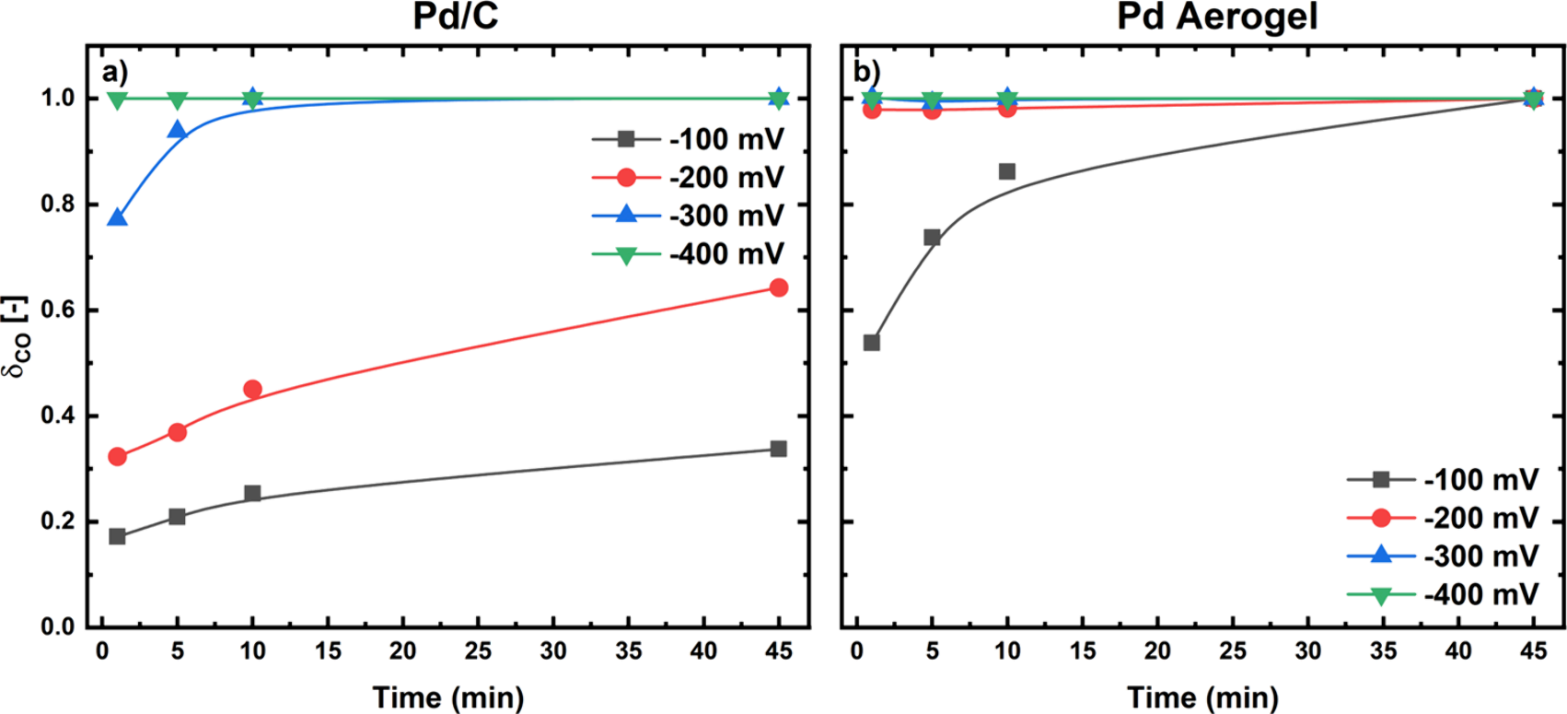
Surface CO coverages calculated from the charges extracted from the CVs shown in Figures S7 and S8 after 1, 5, 10, or 45 min holds at −100, −200, −300, and −400 mV vs RHE in CO_2_-saturated 0.1 M KHCO_3_ for a Pd/C electrode with a loading of 100 μg_Pd_/cm^2^ (a) or a Pd aerogel electrode with a loading of 50 μg_Pd_/cm^2^ (b)

These findings indicate that the Pd aerogel exhibits significantly greater susceptibility to CO poisoning than Pd/C, even at a relatively high potential of – 100 mV vs RHE. Notably, this greater extent of CO-adsorption on the Pd aerogel seems to effectively block the surface sites responsible for the reduction of CO_2_ to formate and hydrogen adsorption, which would explain the low activity for formate production and concomitantly sluggish PdH_*x*_-formation observed for this material as opposed to Pd/C.

To assess whether the increased formate production observed at −200 compared to −100 mV vs RHE for the Pd/C catalyst (see Figure 1b) is solely due to the more negative potential, or rather due to the formation of a more intrinsically active PdH_*x*_ phase, we estimated the ratio between the corresponding exchange current densities (i_0_) at the end of each potential hold (see the ‘Potential- and PdH_*x*_-phase-dependent CO_2_-to-formate activity comparison’ section in the Supporting Information for details). Assuming a cathodic charge transfer coefficient (α_c_) of 0.5 consistent with previous studies,^44,45^ we determined that this ratio between the intrinsic activities at −100 and −200 mV vs RHE (*i*_0,‑100 mV_/*i*_0,‑200 mV_) has a value of 0.38. This clearly indicates that the lower stoichiometric PdH_*x*_ phase formed at -200 mV vs RHE (i.e., with *x* = 0.55 – see Figure 1a) exhibits a ≈2.5-fold higher intrinsic activity toward CO_2_ reduction to formate compared to the phase formed at −100 mV vs RHE (for which *x* = 0.6). This finding supports the hypothesis previously suggested in the literature^6,30^ that mixed α + β PdH_*x*_ phases (like the one observed for Pd/C at −200 mV vs RHE) are more catalytically active for formate production than the fully stoichiometric β PdH_*x*_ counterpart. However, it remains unclear whether this more active, lower-stoichiometry PdH_*x*_ phase solely results from the more negative potential, or if its formation is driven by its increased CO_2_ reduction activity toward formate. More precisely, the latter enhanced activity could lead to a depletion in the concentration of surface-adsorbed hydrogen and corresponding PdH_*x*_ stoichiometry, whereas at potentials of −100 or −300 mV vs RHE entailing a lower catalytic performance, less surface-adsorbed hydrogen is consumed and the β-hydride formation process can be completed (see Figure 1a–c). Beyond this uncertainty, there appears to be an optimal PdH_*x*_ stoichiometry that maximizes the intrinsic catalytic activity toward formate production, and identifying and stabilizing this optimal stoichiometry could be key to further improve the performance of Pd-based electrocatalysts for CO_2_ reduction.

### Assessing the Differences in CO-Adsorption with *Operando* ATR-SEIRAS

To gain a better understanding of the different evolution of the CO adsorption layer on both catalysts in real time, *Operando* ATR-SEIRAS measurements were conducted. The measurements involved again 45 min potential holds within the formate-producing potential regime. Since the working electrode substrate in the ATR-SEIRAS flow cell was replaced with an Au-coated silicon prism (and Au is highly active for CO_2_-to-CO reduction^46^), product quantification was repeated for both catalysts to ensure that the new substrate did not significantly influence the catalytic performance. As shown in Figure S9, both catalysts exhibited CO_2_RR trends that are very similar to those observed with the GIXAS flow cell (cf. Figure 1). Specifically, the Pd/C catalyst demonstrated high pCDs for formate at high potentials (i.e., −100 and −200 mV vs RHE) and fastly decaying formate yields at −300 and −400 mV vs RHE, whereas the Pd aerogel again exhibited low or nonquantifiable pCDs for this product at all studied potentials.

Following this important verification, the time-resolved ATR-SEIRAS spectra recorded at −100, −200, −300, and −400 mV vs RHE are presented in Figure 4, whereby the spectral range focuses on the evolution of two bands corresponding to CO molecules adsorbed on the catalyst surface. The first band between 1990 and 2003 cm^–1^ is attributed to linearly bonded CO molecules (CO_L_), whereas the second and broader one, appearing between 1806 and 1896 cm^–1^, corresponds to bridge- and triple-bonded (also called hollow-bonded) CO molecules (CO_B_ vs CO_T_, respectively). Within this range, CO_T_ species dominate at lower wavenumbers, while CO_B_ species are present at higher wavenumbers.^11,44,47^ Additionally, Figure S10 shows the wavenumber shifts and integral intensities of the band associated with CO_L_ molecules, as well as those of the CO_B_ and CO_T_ molecules together, since their bands are difficult to distinguish, thereby providing further insight into the temporal evolution. It is important to note that the integral intensities should not be directly compared between measurements due to possible variations in sensitivity in between experiments (e.g., due to roughening or delamination of the Au layer in the course of the measurements).^38^

**Figure 4:**
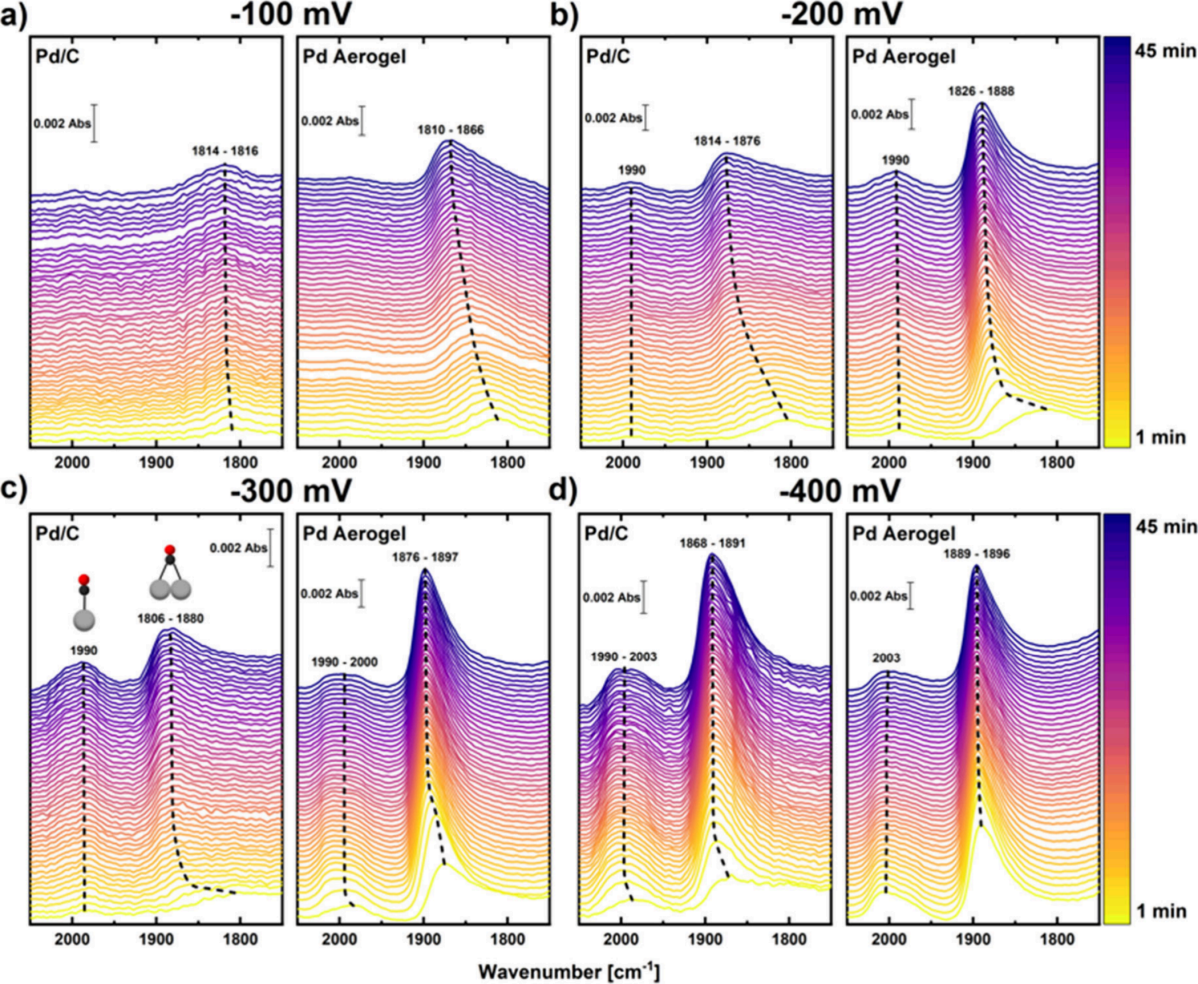
*Operando* ATR-SEIRAS spectra (non-normalized intensities) collected during a 45 min potential holds with a time resolution of ≈1 min per spectrum at −100 (a), −200 (b), −300 (c), or −400 mV vs RHE (d) in CO_2_-saturated 0.1 M KHCO_3_. Left-hand panels display data recorded on a Pd/C electrode with 75 μg_Pd_/cm^2^, while right-hand panels feature the results for a Pd aerogel electrode with a loading of 25 μg_Pd_/cm^2^.

Interestingly, the CO_L_ molecules assignable to a wavenumber shift from 1990 to 2003 cm^–1^ and that are believed to be an intermediate species that desorbs from the catalyst surface to form gaseous CO exhibited a qualitatively similar behavior for both catalysts (see Figures 4 and S10).^47^ However, while the Pd/C catalyst displayed a relatively stable or slightly increasing integral intensity for the CO_L_ band throughout all the potential holds, a decline of these integral signals was observed during the holds at −300 and −400 mV vs RHE for the Pd aerogel (see Figures S10g and S10h). This decrease is likely tied to the onset of gaseous CO production, which possibly remains below the detection limit of the gas-extraction and analytical setup for the hold at −300 mV vs RHE but is unambiguously detected for both catalysts at −400 mV vs RHE (see Figures S9 c and S9f), with the aerogel featuring a ≈2-fold greater CO-pCD consistent with the decreasing integral intensity discussed above.

Focusing on the CO_T+B_ molecules that are generally associated with catalyst poisoning, a notable difference was observed between the two catalysts.^47^ More precisely, the Pd aerogel exhibits a more rapid accumulation of CO_T+B_ on its surface than Pd/C, which is consistent with the stronger CO poisoning discussed in the previous section (see Figures 4 and S10). Nevertheless, this poisoning of the Pd/C catalyst surface in the ATR-SEIRAS configuration seems to occur more gradually than in the GIXAS flow cell, considering the larger formate yields observed for the former experiments throughout the potential hold at −300 mV vs RHE and at the beginning of the hold at −400 mV vs RHE (see Figures 2a vs S9a). This higher formate activity could be caused by the different working electrode substrate and/or by the varying convection properties and corresponding surface reactant concentrations in the two flow cells, whereby the latter property is discussed at length in the text accompanying Figure S2. Beyond these differences, at the higher potentials of −100 mV and −200 mV vs RHE at which the Pd/C catalyst showed a high and quasi-stable activity toward formate, the surface coverage of CO molecules remained low and was dominated by CO_T_ species that are known to be preponderant at low CO-coverages (see Figure 4a and S10a).^48–50^ At more negative potentials and over time, though, CO-poisoning progressed as the CO_T_ molecules rearranged into CO_B_ species due to higher CO coverage, ultimately leading to the strong catalyst poisoning and lack of formate production observed at the end of the potential hold at −400 mV vs RHE.

In the case of the Pd aerogel catalyst, a rapid buildup of CO_B_ species is evident at −200, −300, and −400 mV vs RHE, leading to a wavenumber shift of approximately 1890 cm^–1^ and a fast plateauing in the integral intensity that signals the complete poisoning of the catalyst’s surface at these potentials (see Figures 4 and S10). These observations align with the results for formate production, where detectable amounts were produced only at the beginning of the hold at −200 mV vs RHE and throughout the hold at −100 mV vs RHE (see Figure S9d). Notably, this small extent of formate production at −100 mV is inconsistent with the negligible HCOO^–^-yield observed in the GIXAS cell at the same potential (see Figure 1e), possibly because unlike in that configuration the surface of the Pd aerogel did not get fully poisoned with CO throughout the hold at −100 mV vs RHE in the ATR-SEIRAS cell, as indicated by the fact that the integral intensity of CO_B_ species did not plateau during this measurement (Figure S10a). This discrepancy suggests again that the Au-coated prism substrate and/or differences in cell geometry (Figure S2) may have influenced the catalyst’s behavior. Nonetheless, it remains qualitatively clear that the surface of the Pd aerogel experiences stronger CO-poisoning than that of Pd/C, as proven by the rapid accumulation of CO_B_ species unveiled by these IR-spectroscopy measurements.

### Tying CO_2_RR-Performance to the Catalysts’ Physicochemical Features

To better understand the origin of the catalytic differences between Pd/C and the Pd aerogel, we conducted *ex-situ* pXRD and HAADF-STEM measurements of the two materials. The pXRD diffractograms in Figure 5a feature the characteristic diffraction peaks expected for a face-centered cubic Pd lattice.^9,51^ The primary distinction between the two catalysts lies in the broader peaks for the Pd aerogel compared to Pd/C. Since the nanoparticles in the two catalysts are of comparable size (i.e., those in Pd/C have a diameter of ≈5 nm,^43^ while the Pd aerogel exhibits a web thickness of ≈6 nm^9^), the peak broadening in the aerogel suggests the presence of smaller crystalline domains. In this regard, the HAADF-STEM images of the Pd/C catalyst in Figure 5b reveal that most nanoparticles are composed of single crystalline domains, while the web-like structure of the Pd aerogel in Figure 5c consists of smaller crystalline domains interconnected by an abundance of grain boundaries.^52,53^

**Figure 5:**
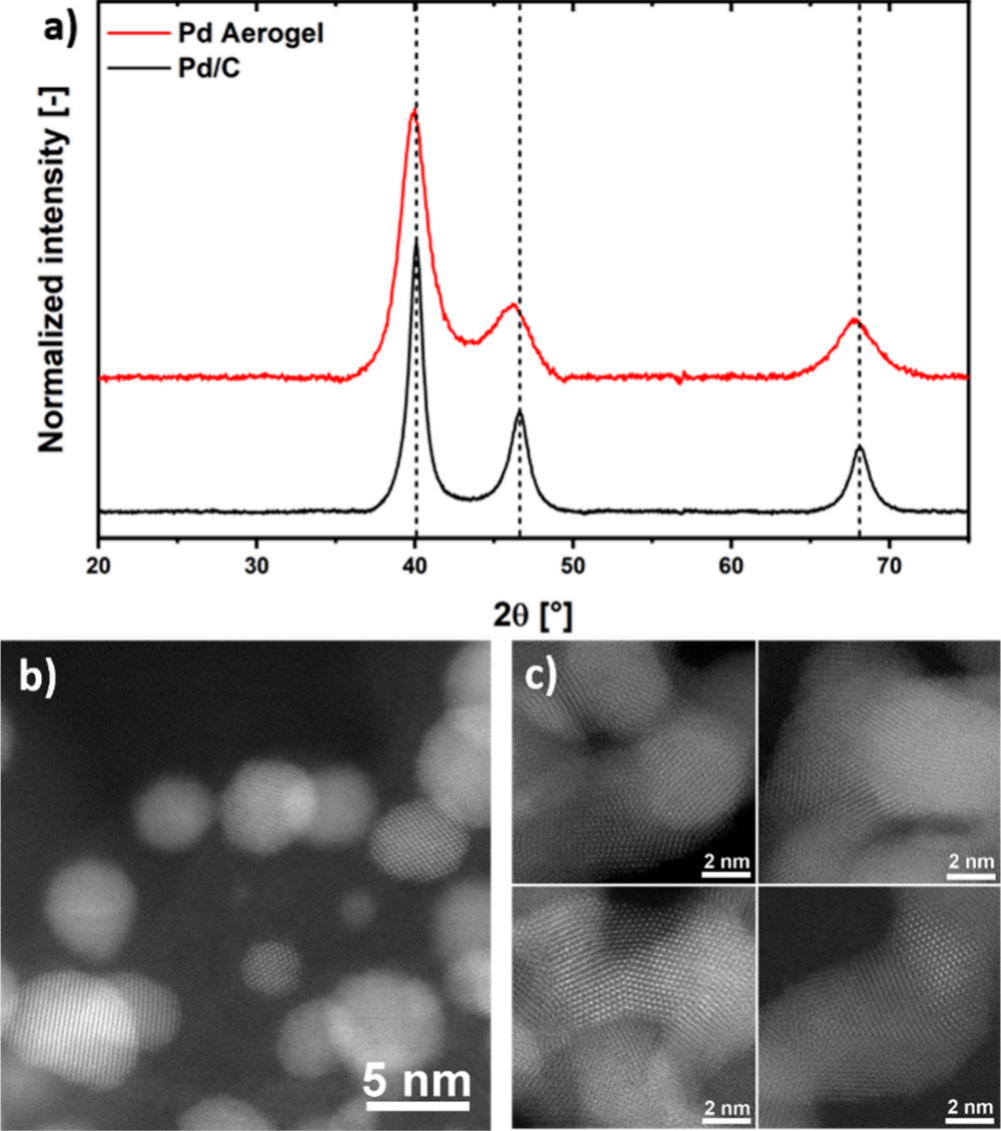
pXRD measurements of the Pd/C and the Pd aerogel (a) and HAADF-STEM images of (b) Pd/C with a predominance of single-crystalline domains, and (c) the Pd aerogel featuring a web-like structure composed of small crystalline domains and abundant grain boundaries.

To tie these observations to the catalysts’ CO_2_RR performance, we start by considering the mechanism of the Pd-catalyzed reduction of CO_2_ to formate, which is generally acknowledged to proceed through the binding of the carbon dioxide molecule to hydrogen adsorbed on the PdH_*x*_-surface (*H), according to the following reaction sequence (in which ‘*’ represents a surface adsorption site.):^7,30,44,45^

H*+CO2→HCOO* 3

HCOO*+e−→*+HCOO− 4

for which the key *HCOO intermediate binds to the PdH_*x*_ surface through *H-populated sites, thus implying that the presence of such surface-adsorbed hydrogen species is a *sine qua non* requisite for formate production. This explains the high CO_2_-to-formate yields featured by the Pd/C surface at potentials of −100 and −200 mV vs RHE at which the Pd/PdH_*x*_ surface is only partially covered with adsorbed CO (see Figures 3a, 4a, and 4b), as schematized in Figure 6, along with the shutdown of the selectivity for formate observed at lower potentials at which the Pd-nanoparticles’ surface is fully CO-poisoned.

**Figure 6:**
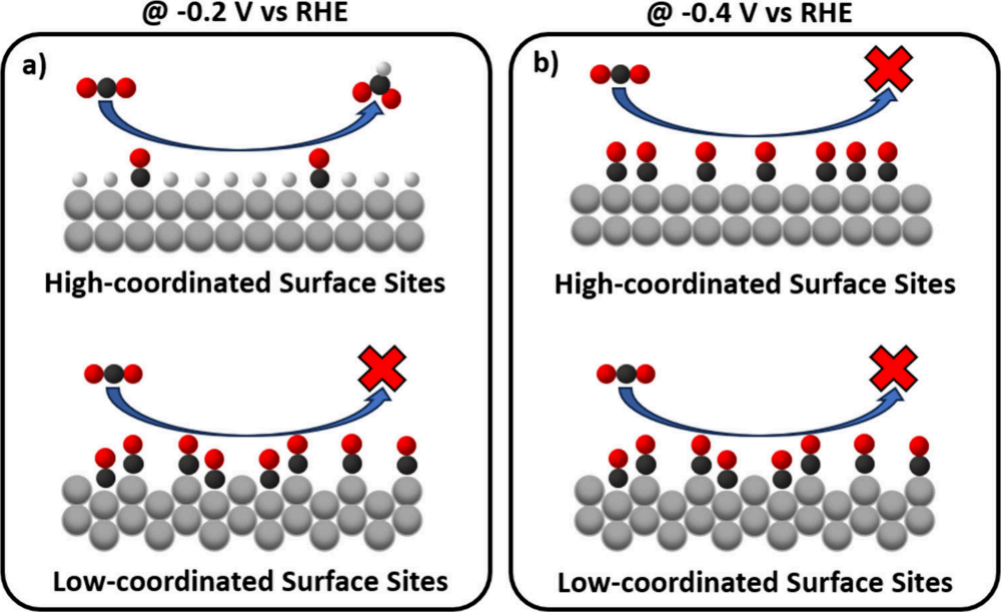
Schematic illustration of the CO_2_RR occurring on a Pd surface with high- and low-coordinated surface sites, represented here as 100 and 110 facets (top vs bottom schemes, respectively) at (a) −0.2 or (b) −0.4 V vs RHE.

As for the Pd-aerogel’s negligible CO_2_-to-HCOO^–^ currents, these can be tied to the (almost) complete CO-coverages and concomitantly negligible availability of *H sites featured by this catalyst at all potentials within the formate-production regime. Notably, this behavior is consistent with the results of recent density functional theory calculations by Abdellah et al.,^30^ who found that the low-coordinated PdH_*x*_-surface domains (likely preponderant in the aerogel’s grain boundaries) have a large affinity for adsorbed CO (consistent with our CO-stripping and IR-spectroscopy results) that renders them inactive for formate production.

Complementarily to this, one can also consider the competing reduction of CO_2_ to gaseous CO on Pd, which again is widely accepted in the literature to proceed through the following reaction steps:

CO2+H++e−+*→COOH* 5

COOH*+H++e−→CO*+H2O 6

CO*→CO+* 7

Among these, the second reaction in which *COOH is converted into *CO occurs very rapidly, while the activation of CO_2_ on the surface to form *COOH and the desorption of CO from the surface have a high energy barrier.^6,11,13,54–57^ Density functional theory (DFT) calculations in the literature^6,55,56^ have proposed that defect sites (such as those featured in grain boundaries) lower the activation barrier for the conversion of CO_2_ to *COOH, thereby further promoting the production of CO molecules adsorbed to the surface—excellently agreeing with the ≈2-fold larger CO_2_-to-CO current featured by the Pd aerogel vs Pd/C at −400 mV vs RHE (see Figure S9c and b) despite the 3-fold lower Pd-loading used for the aerogel electrodes used in those tests.

Thus, even when considering the CO_2_-to-CO mechanism competing with formate production, our spectroelectrochemical results align with our findings from pXRD and HAADF-STEM measurements revealing that the Pd Aerogel exhibits a large number of grain boundaries compared to the single-crystallite Pd/C nanoparticles. More precisely, as schematically showcased in Figure 6, the high density of grain boundaries present in the Pd aerogel leads to the formation of low-coordinated surface sites. These structural features likely facilitate the conversion of CO_2_ to the *COOH intermediate and its subsequent reduction to CO, which covers the catalyst surface and explains the rapid and complete surface poisoning observed for the Pd aerogel catalyst at low overpotentials within the formate-producing potential range. In contrast, this formation of the *COOH intermediate is likely less favorable for the more crystalline surface domains in Pd/C, characterized by high-coordinated surface sites, and that as a result shows a significantly lower susceptibility to CO poisoning at low overpotentials (i.e., −100 and −200 mV vs RHE). This allows the surface to instead be populated by adsorbed hydrogen atoms that enable its high activity toward CO_2_ reduction to formate. At more negative potentials ≤ −300 mV vs RHE, CO formation begins to dominate even on the Pd/C catalyst, leading to surface poisoning and a concurrent decline in formate production. These results imply that the well-known potential-dependent selectivity shift of Pd also has a time-dependent component (stemming from the progressive poising of the surface with CO – see Figures 3 and 4) and is strongly determined by the crystallographic nature of the PdH_*x*_ active phase, since these two features control the progressive accumulation of surface-bound CO that ultimately halts the conversion of CO_2_ into HCOO^–^.

## Conclusion

In summary, in this study we explored how the time-dependent interplay between surface-adsorbed CO and H affect the CO_2_RR to formate on PdH_*x*_ employing *operando* GIXAS and ATR-SEIRAS combined with *online* product analysis. By using two structurally different catalysts (i.e., Pd/C and a Pd aerogel), we were able to directly correlate changes in the state of these materials with their time- and potential-dependent catalytic performance. Specifically, our results revealed that within the formate-producing potential range (−100 to −300 mV vs RHE), the Pd/C catalyst exhibited rapid PdH_*x*_ formation and high formate activity, whereas the Pd aerogel showed sluggish hydride formation and minimal formate production stemming from the rapid poisoning of its surface with CO. The structural characterization of the two materials via HAADF-STEM and pXRD revealed that the Pd aerogel displays a high density of grain boundaries separating smaller crystalline domains, while Pd/C features a more crystalline structure resulting in a surface dominated by high-coordination sites. Based on previous DFT studies, the aerogel’s low-coordinated surface sites promote strong CO adsorption that causes catalyst deactivation, in good agreement with our *operando* IR results. Importantly, we also established that this key role of the CO coverage on the catalytic activity is also time-dependent, as illustrated by our results for Pd/C at −300 mV vs RHE, in which progressive CO poisoning over time led to a gradual decline of the formate yield.

Overall, these insights highlight the need for materials that are resistant to CO poisoning while maintaining an optimal PdH_*x*_ stoichiometry to enable a high formate production activity. Such catalysts could serve as efficient hydrogen donors, thereby improving formate selectivity and the overall CO_2_RR performance. Accordingly, future research should focus on designing nanocatalysts with high-coordinated surface sites, as these structures appear to promote the formation of the HCOO* intermediate through enhanced surface hydrogen coverage. Finally, our combined spectroelectrochemical and analytical approach has been shown to provide a robust framework for probing structure–activity relations that can be extended to the study of this and other electrocatalytic materials and reactions.

## Supplementary Material



## Notes

Additional information on the updated GIXAS flow cell design plus video of the cell assembly, EXAFS fits, oxygen reduction reaction experiment, LSVs after potential holds, product quantification results in ATR-SEIRAS flow cell, additional operando ATR SEIRAS results (PDF)

## ¶

(P.M.L.) Interdisciplinary Nanoscience Center, Aarhus University, 8000 Aarhus C, Denmark

## ○

(M.F.) Bauhaus Luftfahrt e.V., 82024 Taufkirchen, Germany

## △

(T.d.W.) Department of Microsystems Engineering, University of Freiburg, 79110 Freiburg, Germany

## ▼

(M.G.) Infineon Technologies Dresden GmbH & Co. KG, 01099 Dresden, Germany

## Abbreviations

CO_2_RR: Carbon Dioxide Reduction Reaction
Pd: Palladium
GIXAS: Grazing Incidence X-ray Absorption Spectroscopy
ATR-SEIRAS: Attenuated Total Reflectance Surface-Enhanced Infrared Absorption Spectroscopy
ECSA: Electrochemically Active Surface Area
HAADF-STEM: High-Angle Annular Dark-Field Scanning Transmission Electron Microscopy
pXRD: Powder X-ray Diffraction
DFT: Density Functional Theory
RHE: Reversible Hydrogen Electrode
EXAFS: Extended X-ray Absorption Fine Structure
LSV: Linear Sweep Voltammetry
CV: Cyclic Voltammetry
PdH_*x*_: Palladium Hydride
CO: Carbon Monoxide
