# Correction to “Mechanistic Insights into the Electroreduction of Carbon Dioxide to Formate on Palladium”

**DOI:** 10.1021/acscatal.5c08966

**Published:** 2026-01-13

**Authors:** Maximilian Winzely, Deema Balalta, Adam H. Clark, Tommaso Iarocci, Alexandr Oshchepkov, Paul M. Leidinger, Davide Masiello, Meriem Fikry, Tym de Wild, Maximilian Georgi, Sara Bals, Thomas J. Schmidt, Juan Herranz

Alexandr Oshchepkov has been added as an author of this paper, since he played a key role in the completion of the *in situ* IR-spectroscopy measurements featured in the manuscript.

