# Supplementary material for: Mechanistic Insights into the Electroreduction of Carbon Dioxide to Formate on Palladium
Source: ACS Catal. 2025 Sep 25;15(20):17065–77. doi: 10.1021/acscatal.5c04052 (PMC12538717; doi:10.1021/acscatal.5c04052)
Supplement: Supplementary file 1 [file cs5c04052_si_001.pdf]

Supporting Information

Mechanistic Insights into the Electroreduction of Carbon Dioxide to Formate on Palladium

Maximilian Winzely[1], Deema Balalta[2], Adam H. Clark,[3] Tommaso Iarocci[1], Paul M. Leidinger[1], Davide Masiello[1], Meriem Fikry[1], Tym de Wild[1], Maximilian Georgi[4], Sara Bals[2], Thomas J. Schmidt[1,5], Juan Herranz[1*]

[1] PSI Center for Energy and Environmental Science, CH-5232 Villigen PSI, Switzerland

[2] University of Antwerp, Electron Microscopy for Materials Science, BE-2020 Antwerpen, Belgium

[3] PSI Center for Photon Science, CH-5232 Villigen PSI, Switzerland

[4] Technische Universität Dresden, Physical Chemistry, DE-01062 Dresden, Germany

[5] ETH Zürich, Institute for Molecular Physical Science, CH-8093 Zürich, Switzerland

[*]Corresponding author: juan.herranz@psi.ch

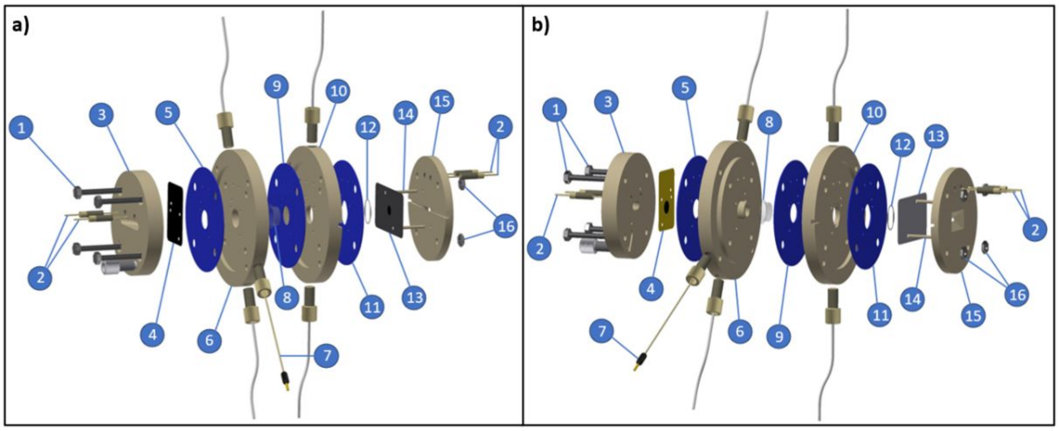

Figure S1. Technical sketch of the GIXAS cell assembly from two different view angles a) and b) consisting of four M4 screws (1), four gold contact pins (2), the outer counter-electrode part (3), the counter electrode (4), the counter-electrode gasket (5), the inner counter-electrode part (6), leak-free Ag/AgCl reference electrode (7), a piece of Nafion HP membrane (8), membrane gaskets (9), the inner working electrode part (10), the working electrode gasket (11), Teflon flow field (12), the working electrode (i.e., a piece of graphene sheet and catalyst 13), alignment pins (14), the outer working electrode part (15), and nuts (16).

Oxygen Reduction Reaction (ORR) experiment

To assess the mass transport properties of the updated flow field, ORR experiments were conducted on a Pt working electrode. A 100 nm thick polycrystalline Pt layer was sputtered onto a specialized carbon-coated Kapton foil (electrically conductive Kapton 200RS100 from DuPont) in the area designated for catalyst drop-casting. The experiments were performed using 0.1 M $HClO_4$, prepared by diluting 70 wt.% concentrated $HClO_4$ (Merck, Suprapur®) with ultrapure water.

The electrolyte was first saturated with Ar gas for approximately 15 minutes, after which the cell was filled with the electrolyte at a flow rate of 0.5 mL/min. The initial electrochemical measurement involved

recording an impedance spectrum using a potential perturbation of 10 mV, yielding a resistance value of approximately 22 Ω·cm². All subsequent potentials were corrected by accounting for 85% of this resistance value. To condition the Pt electrode, cyclic voltammetry was performed at a scan rate of 100 mV/s over a potential range of 0.03 to 1.45 V versus RHE until a stable current profile was achieved. A baseline CV was then recorded at a reduced scan rate of 50 mV/s within the same potential range.

Subsequently, the electrolyte was saturated with O_2 gas for 15 minutes, and CVs were recorded at varying flow rates between 0.5 and 2 mL/min to determine the limiting current for the ORR at each flow rate. CV measurements were performed at 50 mV/s over the same potential range until a stable current profile was observed, as shown in Figure S2a. The diffusion boundary layer (DBL) thickness was calculated using the following equation:

$$DBL\ thickness = \frac{c_{O2_{bulk}} * D_{O2} * n * F}{i_{lim}} (1)$$

whereby c_{O2_bulk} is the concentration of oxygen in the bulk of the electrolyte ($1.39 \cdot 10^{-3}$ mol/L at 25 °C) [1], D_{O2} is the diffusion constant of oxygen in water ($2.42 \cdot 10^{-5}$ cm^2/s at 25 °C) [1], n are the number of electrons, F is the Faraday constant and i_{lim} is the limiting current density at each flow rate. The limiting current density was determined by averaging the current density between 0.4 and 0.46 V versus RHE in both the positive and negative scan directions.

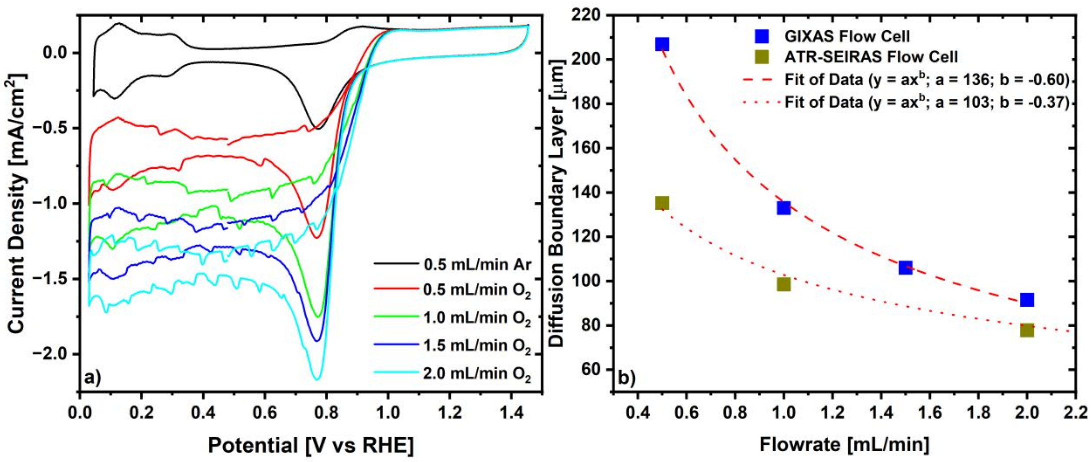

Figure S2. (a) CVs recorded for a 100 nm thick polycrystalline Pt working electrode in 0.1 M $HClO_4$ under N_2- and O_2-saturated conditions at a scan rate of 50 mV/s. For the O_2-saturated electrolyte, flow rates ranged from 0.5 to 2 mL/min. (b) DBL thicknesses calculated (blue squares) from the limiting current densities in panel (a) using Equation 1. As comparison the DBL thicknesses for the ATR-SEIRAS flow cell are shown with the green squares.

The calculated DBL thicknesses are presented in Figure 2b and demonstrate comparable results to those obtained using the ATR-SEIRAS flow cell [2] which is also employed in this study. Specifically, at a flow rate of 2 mL/min, the DBL thicknesses were 92 µm and 78 µm for the updated flow cell and the ATR-SEIRAS flow cell, respectively, indicating that both flow cells exhibit similarly efficient mass transport properties.

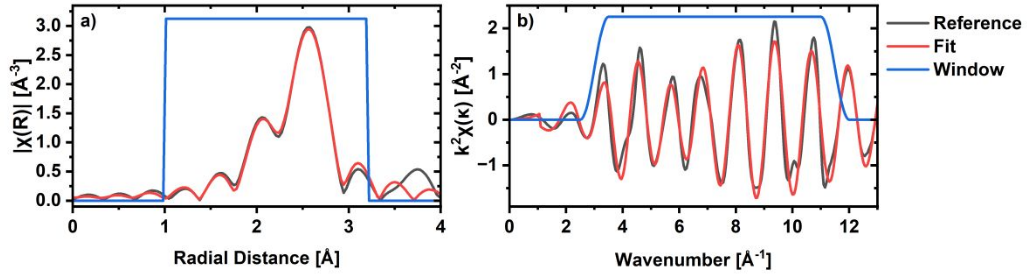

Figure S3. k^2-space and R-space EXAFS fits at the Pd K-edge for the Pd reference foil used in energy calibration. The fitting was performed over a k-range of 3 to 11.5 $Å^{-1}$. Both panels display the data (black line), the fit (red line), and

the Hanning fitting window (blue line), with the left panel showing the k^2-space and the right panel showing the R-space results.

Table S1. Fitting parameters of the EXAFS fit (shown in Figure S2) of the Pd reference foil used for energy calibration.

CN_{Pd} [-]	S_0^2	σ^2 [10^{-3}Å^{-2}]	ΔE_0 [eV]	R_{Pd} [Å]
12	0.770 ± 0.038	5.14 ± 0.03	4.34 ± 0.36	2.735 ± 0.002

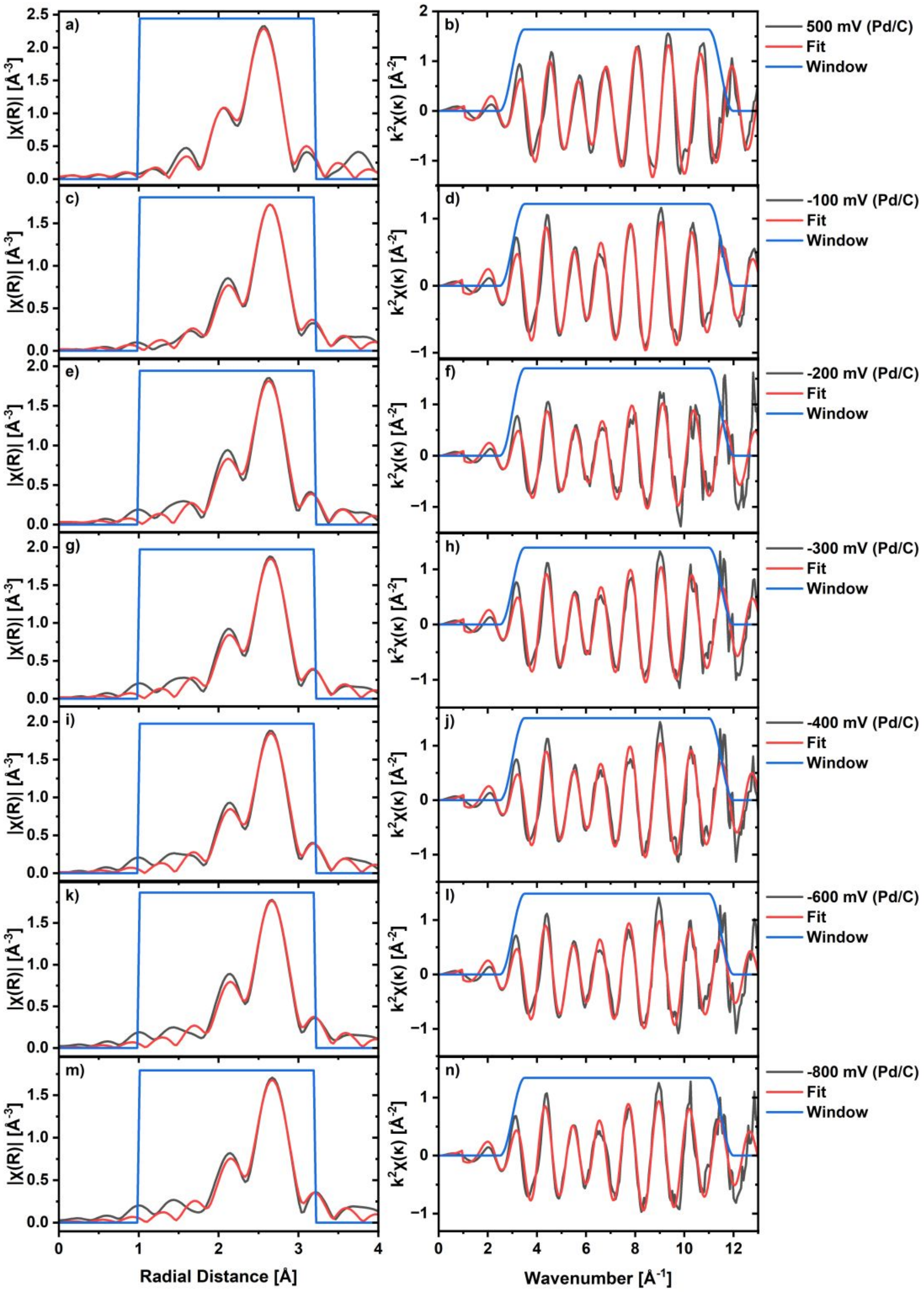

Figure S2. k²-space and R-space EXAFS fits at the Pd K-edge for a Pd/C working electrode with a loading of 100 $\mu g_{Pd}/cm^2$ in CO_2-saturated 0.1 M $KHCO_3$. Each fit corresponds to the final 5 minutes of a 45-minute potential hold, except for the 0.5 V vs RHE hold, which represents a 10-minute hold. All panels display the data (black line),

the fit (red line), and the Hanning fitting window (blue line), with the left panels showing the k^2-space and the right panels showing the R-space results.

Table S2. Fitting parameters of the EXAFS fit (shown in Figure S4) of the Pd/C working electrode with a loading of 100 $\mu g_{Pd}/cm^2$ in CO_2-saturated 0.1 M $KHCO_3$. Each fit corresponds to the final 5 minutes of a 45-minute potential hold, except for the 0.5 V vs RHE hold, which represents a 10-minute hold.

**Potential [V_{RHE}]**	**CN_{Pd} [-]**	**σ^2 [10^{-3}Å^{-2}]**	**ΔE_0 [eV]**	**R_{Pd} [Å]**
0.5	9.46 ± 0.76	5.27 ± 0.52	4.39 ± 0.58	2.735 ± 0.003
-0.1	9.59 ± 0.64	6.94 ± 0.47	3.45 ± 0.47	2.820 ± 0.003
-0.2	9.06 ± 1.34	6.21 ± 1.01	3.85 ± 1.04	2.802 ± 0.007
-0.3	9.77 ± 1.31	6.50 ± 0.92	4.01 ± 0.93	2.823 ± 0.006
-0.4	9.55 ± 1.28	6.29 ± 0.91	4.00 ± 0.93	2.828 ± 0.006
-0.6	9.96 ± 1.26	6.88 ± 0.89	3.70 ± 0.87	2.840 ± 0.006
-0.8	9.48 ± 1.34	6.83 ± 0.99	3.59 ± 0.98	2.846 ± 0.007

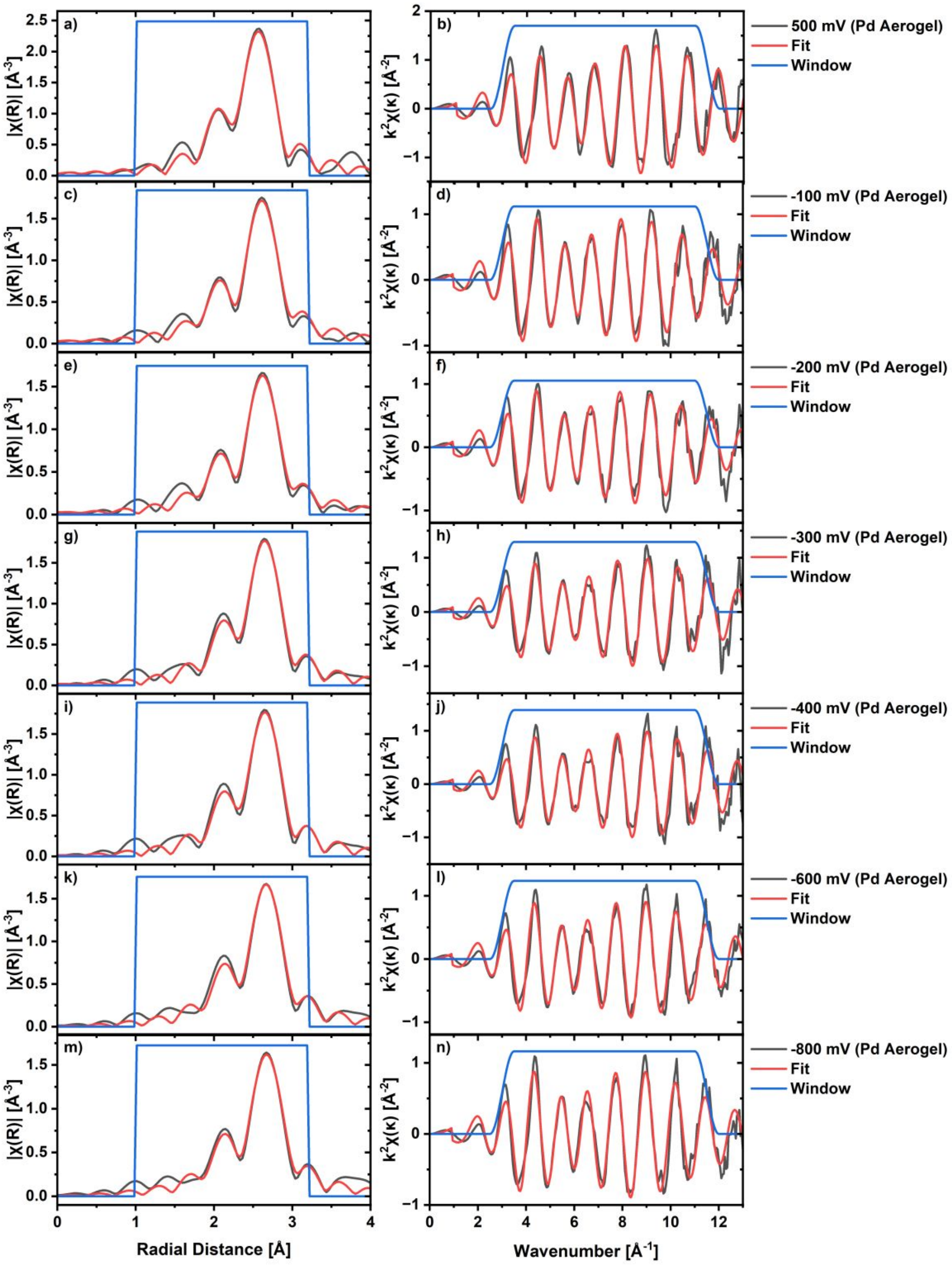

Figure S3. k²-space and R-space EXAFS fits at the Pd K-edge for a Pd Aerogel working electrode with a loading of 50 $\mu g_{Pd}/cm^2$ in CO_2-saturated 0.1 M $KHCO_3$. Each fit corresponds to the final 5 minutes of a 45-minute potential hold, except for the 0.5 V vs RHE hold, which represents a 10-minute hold. All panels display the data (black line), the fit (red line), and the Hanning fitting window (blue line), with the left panels showing the k²-space and the right panels showing the R-space results.

Table S3. Fitting parameters of the EXAFS fit (shown in Figure S5) of the Pd Aerogel working electrode with a loading of 50 $\mu g_{Pd}/cm^2$ in CO_2-saturated 0.1 M $KHCO_3$. Each fit corresponds to the final 5 minutes of a 45-minute potential hold, except for the 0.5 V vs RHE hold, which represents a 10-minute hold.

**Potential [V_{RHE}]**	**CN_{Pd} [-]**	**σ^2 [10^{-3}Å^{-2}]**	**ΔE_0 [eV]**	**R_{Pd} [Å]**
0.5	10.6 ± 1.0	6.08 ± 0.66	4.25 ± 0.69	2.737 ± 0.004
-0.1	10.3 ± 1.1	7.95 ± 0.76	3.71 ± 0.71	2.778 ± 0.005
-0.2	10.0 ± 1.2	8.03 ± 0.91	3.51 ± 0.84	2.790 ± 0.006
-0.3	9.78 ± 1.16	6.85 ± 0.83	3.57 ± 0.82	2.822 ± 0.006
-0.4	9.58 ± 1.25	6.67 ± 0.91	3.60 ± 0.91	2.826 ± 0.006
-0.6	10.1 ± 1.1	7.48 ± 0.82	3.43 ± 0.78	2.840 ± 0.006
-0.8	10.2 ± 1.1	7.72 ± 0.76	3.29 ± 0.71	2.846 ± 0.005

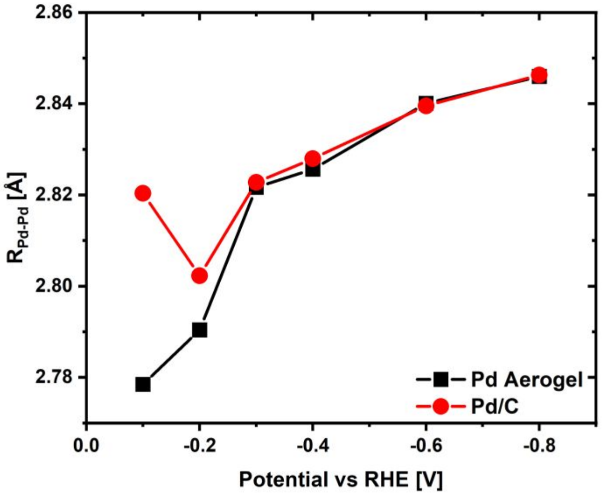

Figure S4. Interatomic bonding distances extracted from EXAFS fits (see Figure S4 and S5; Table S2 and S3) for a Pd/C working electrode with a loading of 100 $\mu g_{Pd}/cm^2$ and a Pd Aerogel working electrode with a loading of 50 $\mu g_{Pd}/cm^2$ in CO_2-saturated 0.1 M $KHCO_3$. Each datapoint represent the last 5 minutes of a 45-minute potential hold.

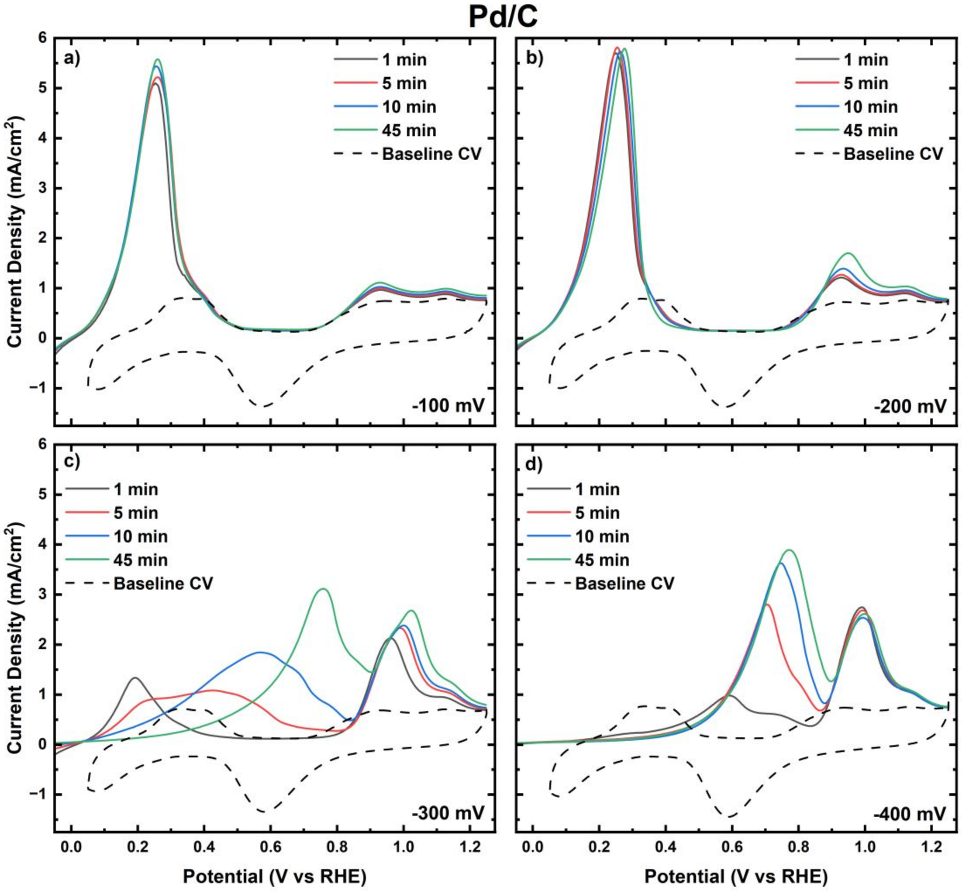

Figure S5. LSVs recorded at a scan rate of 20 mV/s following potential holds of 1, 5, 10, and 45 minutes at a) –100 mV, b) –200 mV, c) –300 mV and d) –400 mV vs RHE. Measurements were performed in CO_2-saturated 0.1 M $KHCO_3$, starting from the holding potential and extending to 1.25 V vs RHE using a Pd/C working electrode with a loading of 100 $\mu g_{Pd}/cm^2$. A baseline CV of the working electrode, recorded over the potential range of 0.05 to 1.25 V vs RHE, is depicted as a black dashed line.

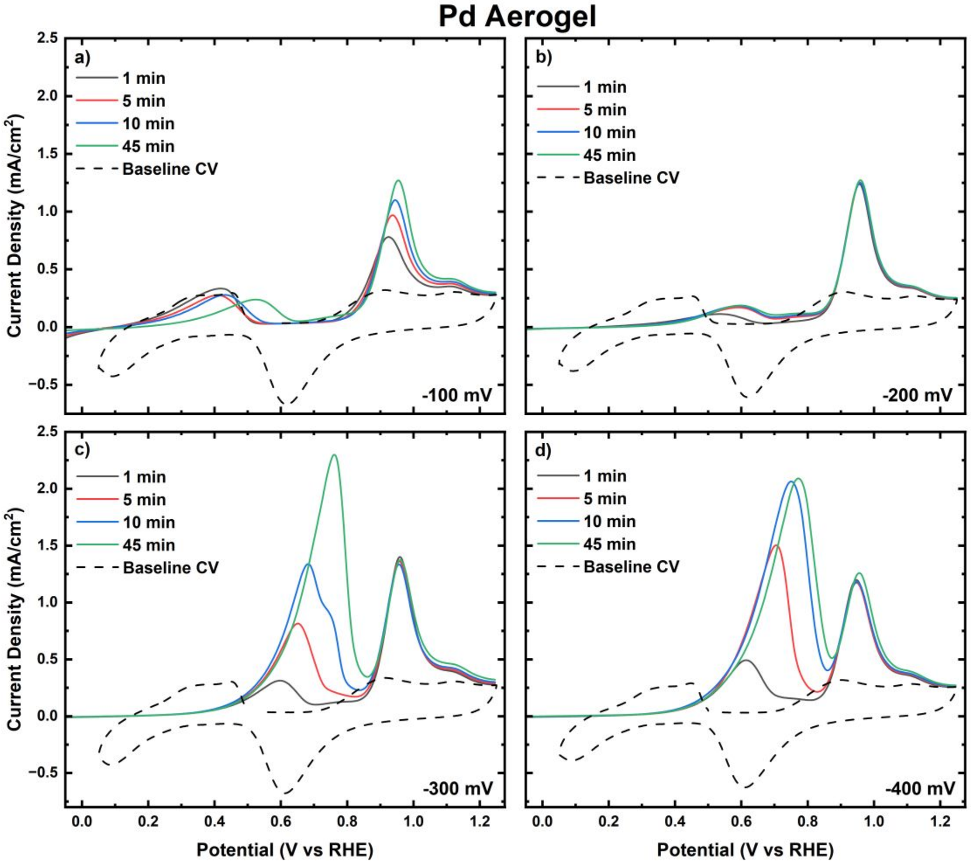

Figure S6. LSVs recorded at a scan rate of 20 mV/s following potential holds of 1, 5, 10, and 45 minutes at a) –100 mV, b) –200 mV, c) –300 mV and d) –400 mV vs RHE. Measurements were performed in CO_2-saturated 0.1 M $KHCO_3$, starting from the holding potential and extending to 1.25 V vs RHE using a Pd Aerogel working electrode with a loading of 50 $\mu g_{Pd}/cm^2$. A baseline CV of the working electrode, recorded over the potential range of 0.05 to 1.25 V vs RHE, is depicted as a black dashed line.

Potential- and PdH_x-phase-dependent CO_2-to-formate activity comparison

To evaluate whether the intrinsic CO_2-to-formate activity of the catalysts at different potentials differs depending on the corresponding PdH_x phases formed, we compared the cathodic branch of the Butler–

Volmer equation, which relates the kinetically-controlled current to the corresponding potential. We begin by expressing these cathodic currents (*i*s) at two different potentials, E_1 and E_2, as follows:

$$i(E_1, t) = i_{0,1} \cdot L \cdot ECSA \cdot \left[1 - \theta_{CO}(E_1, t)\right] \cdot e^{-\frac{\alpha_c F}{RT}(E_1 - E_{rev})} (2)$$

$$i(E_2, t^*) = i_{0,2} \cdot L \cdot ECSA \cdot \left[1 - \theta_{CO}(E_2, t^*)\right] \cdot e^{-\frac{\alpha_c F}{RT}(E_2 - E_{rev})} (3)$$

where $i_{0,1}$ and $i_{0,2}$ are the exchange current densities at E_1 and E_2, respectively, *L* is the catalyst loading, ESCA is the electrochemical surface area, θ_{CO} is the time- and potential-dependent CO surface coverage, α_c is the cathodic charge transfer coefficient (assumed to be 0.5 [3,4]), *F* is the Faraday constant, *T* is the temperature and E_{rev} is the reversible potential of the reaction.

To extract the ratio of the intrinsic activities at the two potentials (i.e., the exchange current densities' quotient, $i_{0,1}/ i_{0,2}$), we divide Equation 2 by Equation 3:

$$\frac{i(E_1, t)}{i(E_2, t^*)} = \frac{i_{0,1}}{i_{0,2}} \cdot \frac{1 - \theta_{CO}(E_1, t)}{1 - \theta_{CO}(E_2, t^*)} \cdot e^{-\frac{\alpha_c F}{RT}(E_1 - E_2)} (4)$$

Rearranging Equation 4 yields the expression for the ratio of the two exchange current densities:

$$\frac{i_{0,1}}{i_{0,2}} = \frac{i(E_1, t)}{i(E_2, t^*)} \cdot \frac{1 - \theta_{CO}(E_2, t^*)}{1 - \theta_{CO}(E_1, t)} \cdot e^{\frac{\alpha_c F}{RT}(E_1 - E_2)} (5)$$

This formulation allows us to isolate the change in intrinsic activity (via i_0) while correcting for differences in CO poisoning (via θ_{CO}). By using the values presented in Table S4, which describe the state of the formate activity and CO poisoning of the Pd/C catalysts at the end of the potential hold at -100 and -200 mV vs RHE a ratio for $i_{0,-100\ mV}/i_{0,-200\ mV}$ of 0.38 was calculated.

Table S4. Values for θ_{CO} and pCD for formate recorded on a Pd/C working electrode with a loading of 100 $\mu g_{Pd}/cm^2$ after 45 minutes of potential holds at -100 or -200 mV vs RHE in CO_2-saturated 0.1 M $KHCO_3$.

Potential [mV vs RHE]	Θ_{CO}	$pCD_{Formate}$ [mA/cm^2]
-100	0.34	-0.2
-200	0.64	-2.1

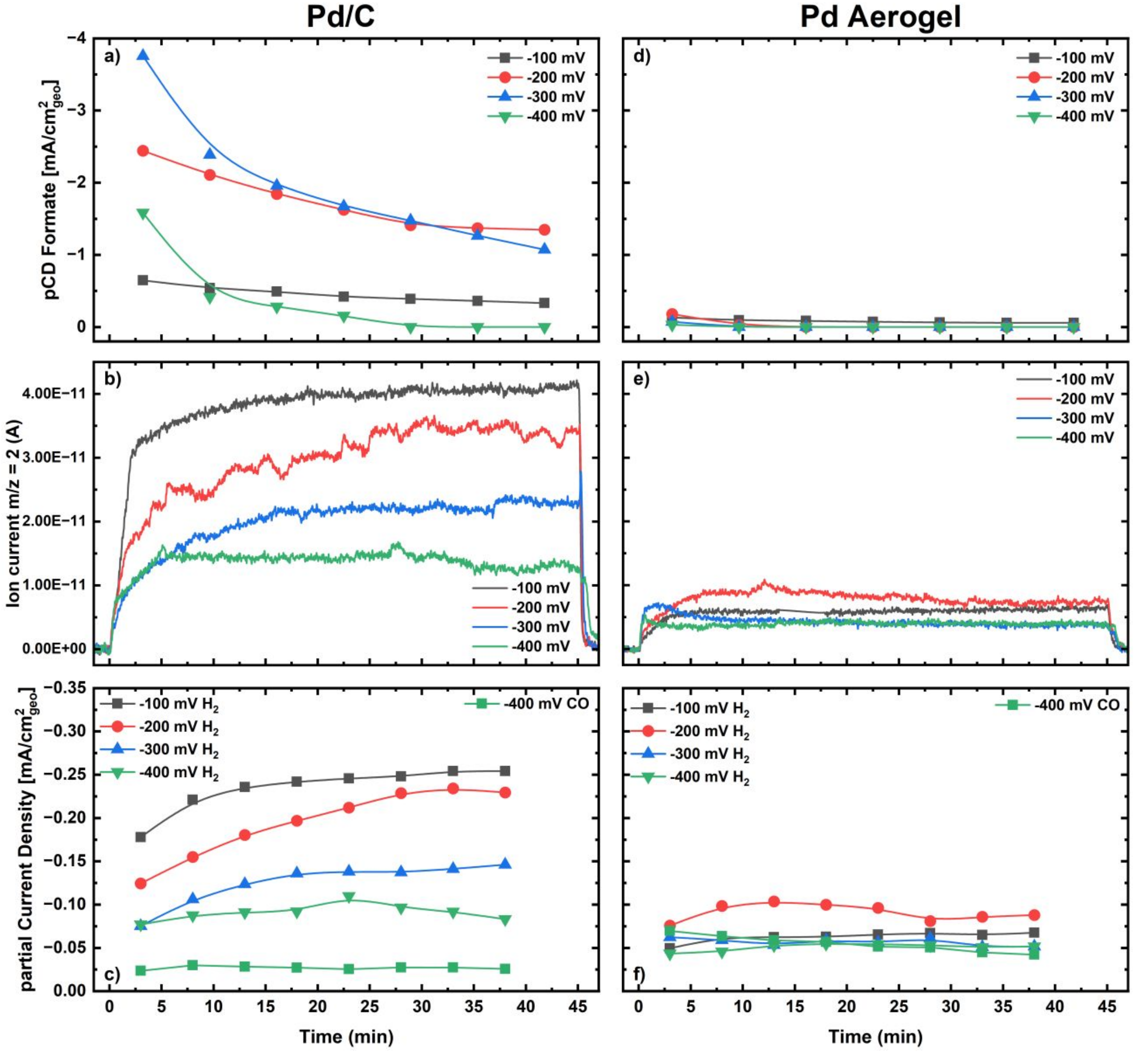

Figure S7. (a) Partial current density (pCD) for formate, (b) ion current signal from the mass spectrometer for m/z = 2, and (c) pCD for H_2 and CO, recorded for a Pd/C working electrode with a Pd loading of 75 $\mu g_{Pd}/cm^2$ in CO_2-saturated 0.1 M $KHCO_3$ during a 45-minute potential hold at –100, –200, –300, and –400 mV vs RHE. (d) pCD for formate, (e) ion current signal from the mass spectrometer for m/z = 2, and (f) pCD for H_2 and CO, recorded for a Pd aerogel working electrode with a Pd loading of 25 $\mu g_{Pd}/cm^2$ in CO_2-saturated 0.1 M $KHCO_3$ during a 45-minute potential hold at –100, –200, –300, and –400 mV vs RHE.

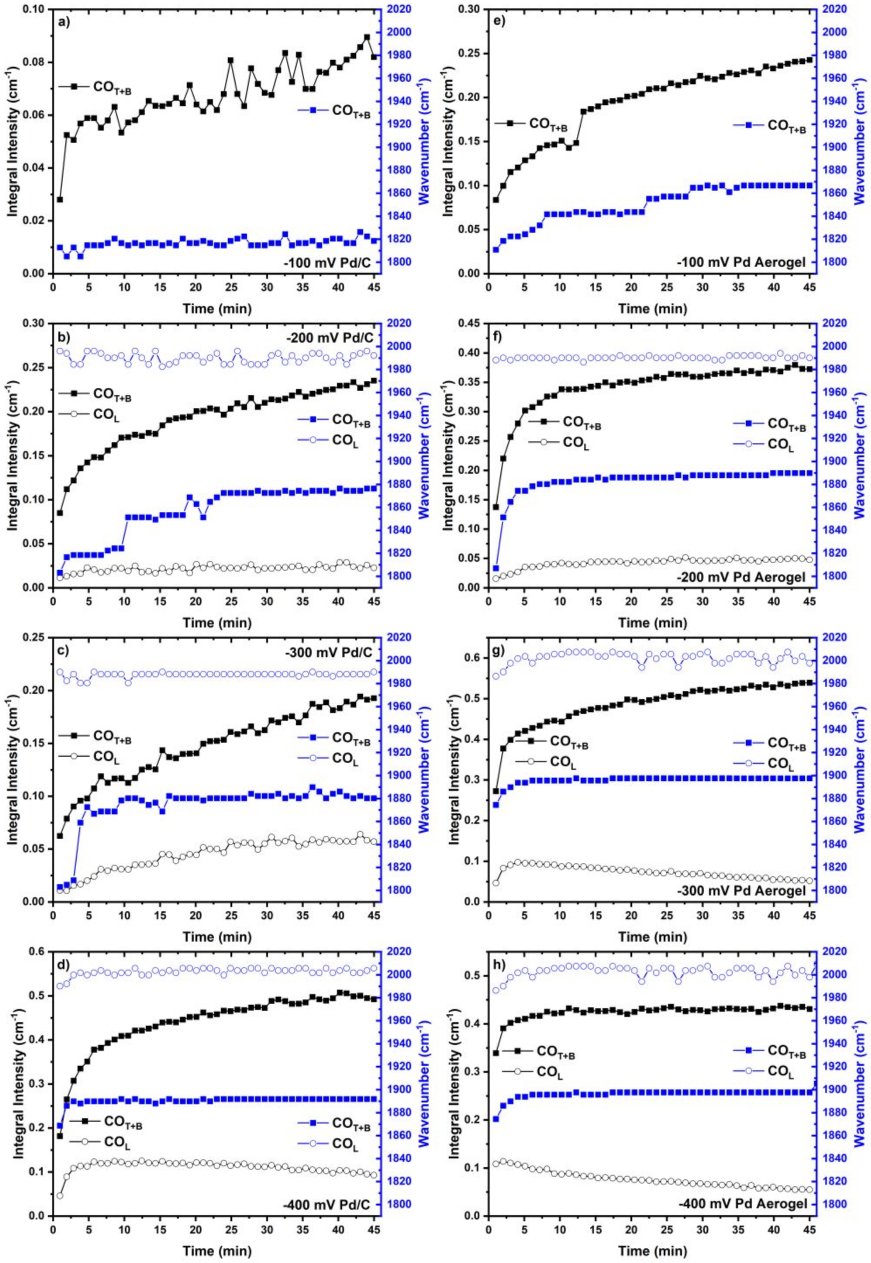

Figure S8. Integral intensities and wavenumber shifts of CO_L and CO_{T+B} bands of the operando ATR-SEIRAS measurements shown in Figure 4 for a Pd/C working electrode with a loading of 75 μg$_{Pd}$/cm² in CO_2-saturated 0.1 M $KHCO_3$ during a 45-minute potential hold at a) –100, b) –200, c) –300, and d) –400 mV vs RHE and for a Pd aerogel working electrode with a Pd loading of 25 μg$_{Pd}$/cm² in CO_2-saturated 0.1 M $KHCO_3$ during a 45-minute potential hold at e) –100, f) –200, g) –300, and h) –400 mV vs RHE.

References

(1) Lide, D. R. *CRC handbook of chemistry and physics*; CRC press, 2004.

(2) Winzely, M.; Linke, J.; Oshchepkov, A.; Rüttimann, P.; Welmers, T.; Fabbri, E.; Ferri, D.; Schmidt, T. J.; Juan, H. A spectroelectrochemical Flow Cell Setup for operando ATR-SEIRAS Investigations combined with online Reaction Product Quantification. *submitted* **2025**.
